# Developmentally regulated mitochondrial biogenesis and cell death competence in maize pollen

**DOI:** 10.1186/s12870-022-03897-y

**Published:** 2022-11-01

**Authors:** Karen C. Chamusco, May N. Milazzo, Kanchan S. Bhan, Terry L. Kamps, Prestina Smith, Modupeoluwa Durojaiye, Cristina D. Moreira, Maria Gallo, Christine D. Chase

**Affiliations:** 1 Horticultural Sciences Department, University of Florida, Gainesville, FL 32611-0690 USA; 2 Emergency Department, Baton Rouge General Medical Center, Baton Rouge, LA 70809 USA; 3 Department of Plant Molecular Biology and Biotechnology, Indira Gandhi Agricultural University, Raipur, C.G. 492012 India; 4 Division of Pulmonary, Allergy, Critical Care and Sleep Medicine, Emory University School of Medicine, Atlanta, GA 30322 USA; 5 Department of Family and Community Medicine, University of Cincinnati College of Medicine, Cincinnati, OH 45267 USA; 6 Department of Biology, University of North Carolina Greensboro, Greensboro, NC 27412 USA; 7 Department of Plant and Earth Science, University of Wisconsin-River Falls, River Falls, WI 54022 USA

**Keywords:** Maize, Mitochondria, Pollen development, Cytoplasmic male sterility, Programmed cell death

## Abstract

**Background:**

Cytoplasmic male sterility (CMS) is a maternally inherited failure to produce functional pollen that most commonly results from expression of novel, chimeric mitochondrial genes. In *Zea mays*, cytoplasmic male sterility type S (CMS-S) is characterized by the collapse of immature, bi-cellular pollen. Molecular and cellular features of developing CMS-S and normal (N) cytoplasm pollen were compared to determine the role of mitochondria in these differing developmental fates.

**Results:**

Terminal deoxynucleotidyl transferase dUTP nick end labeling revealed both chromatin and nuclear fragmentation in the collapsed CMS-S pollen, demonstrating a programmed cell death (PCD) event sharing morphological features with mitochondria-signaled apoptosis in animals. Maize plants expressing mitochondria-targeted green fluorescent protein (GFP) demonstrated dynamic changes in mitochondrial morphology and association with actin filaments through the course of N-cytoplasm pollen development, whereas mitochondrial targeting of GFP was lost and actin filaments were disorganized in developing CMS-S pollen. Immunoblotting revealed significant developmental regulation of mitochondrial biogenesis in both CMS-S and N mito-types. Nuclear and mitochondrial genome encoded components of the cytochrome respiratory pathway and ATP synthase were of low abundance at the microspore stage, but microspores accumulated abundant nuclear-encoded alternative oxidase (AOX). Cytochrome pathway and ATP synthase components accumulated whereas AOX levels declined during the maturation of N bi-cellular pollen. Increased abundance of cytochrome pathway components and declining AOX also characterized collapsed CMS-S pollen. The accumulation and robust RNA editing of mitochondrial transcripts implicated translational or post-translational control for the developmentally regulated accumulation of mitochondria-encoded proteins in both mito-types.

**Conclusions:**

CMS-S pollen collapse is a PCD event coincident with developmentally programmed mitochondrial events including the accumulation of mitochondrial respiratory proteins and declining protection against mitochondrial generation of reactive oxygen species.

**Supplementary Information:**

The online version contains supplementary material available at 10.1186/s12870-022-03897-y.

## Background

Cytoplasmic male sterility (CMS) is a maternally inherited failure to produce functional pollen that commonly results from expression of novel, chimeric mitochondrial genes [1]. Mitochondrial CMS genes alter mitochondrial functions to result in either the degeneration or homeotic transformation of male reproductive organs (reviewed by [2–6]). In some examples, male reproductive organs (stamens) are transformed into petals or carpels [7–11]. Other examples are characterized by the degeneration of anther tissues and/or the developing pollen. In the PET-1 CMS of sunflower, premature death of the tapetal cells lining the anther is characterized by features shared with apoptotic programmed cell death (PCD) events in animals [12], characterized as death with shrinkage [13]. In CMS-T maize, the tapetal cells exhibit features of oncotic cell death [14], characterized as death with swelling [15]. The molecular and cellular events that culminate in these diverse CMS phenotypes are of interest and importance in understanding mitochondrial functions that support normal plant reproduction and productivity [6, 16].

PCD can occur by a diversity of pathways and contributes to normal patterns of growth and development and to the disease and defense responses of both plants [17–20] and animals [21, 22]. Mitochondria are well-characterized signaling components of apoptotic PCD pathways in animals [21] and there is evidence of mitochondrial involvement in the plant pathways to PCD [15, 17, 19, 23, 24]. Despite conservation in function and importance, there is limited conservation between the PCD executors of plants and animals [15, 23, 25–28]. CMS systems that culminate in cell death programs provide an additional link between plant mitochondria and PCD, and offer a means to investigate mitochondrial cell death signals and down-stream targets in plants [12, 29].

CMS-S maize is a gametophytic system [30] wherein pollen expressing the mitochondrial CMS-S locus collapses following the microspore (MSP) mitosis [31]. This cell division marks the transition between the uni-nucleate MSP and bi-cellular pollen (BCP) stages of development [32]. The morphological features of collapsed CMS-S pollen, including condensed cytoplasm with numerous membrane-bound packets and membranous whorls [31], are similar to those of animal cells undergoing apoptotic PCD [13, 33]. Maize plants produce abundant pollen, and the uni-nucleate MSPs can be physically separated from BCP on sucrose density gradients [34, 35]. Here we exploit these features to demonstrate an unexpected developmental regulation of mitochondrial biogenesis during maize pollen development, concomitant with actuation of PCD in the CMS-S mito-type.

## Results

### S-cytoplasm pollen collapse is a programmed cell death event

**Fig. 1:**
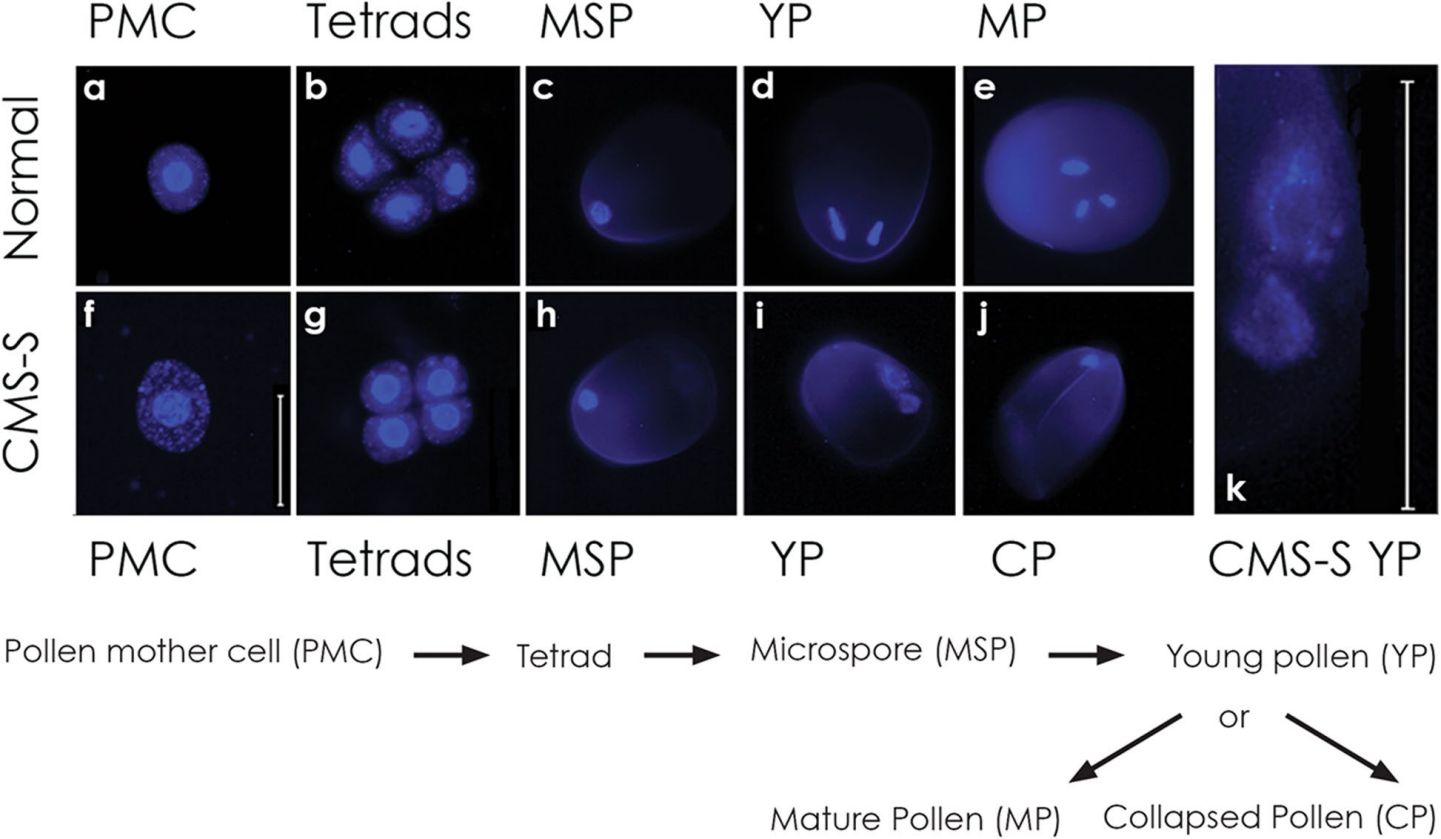
Normal (N) and CMS-S pollen development visualized with DAPI stain. A flow diagram of pollen development is presented beneath DAPI-stained images of key developmental stages. **a-c** N-cytoplasm and **f–h** CMS-S pollen appearing morphologically identical at the **a**, **f** pollen mother cell (PMC); **b**, **g** tetrad; and **c**, **h** microspore (MSP) stages. **d**, **i** Young bi-cellular pollen (YP) with one nucleus fragmenting in CMS-S. **e** N-cytoplasm, mature, tri-cellular pollen (MP). **j** Collapsed, bi-cellular CMS-S pollen (CP). **k** Enlarged detail of nuclei from panel **i** showing marginalized, fragmented chromatin. Bars = 50 µm

The DNA-binding dye 4', 6-Diamidino-2-phenylindole (DAPI) was used to visualize and compare nuclear features of CMS-S and normal (N) cytoplasm maize pollen development (Fig. 1). The two mito-types shared similar cytological features through the MSP stage (Fig. 1a-c, f–h). In addition to DAPI stained nuclei, strong punctate, cytosolic signals are seen in the pollen mother cell (PMC) and tetrad stages (Fig. 1a,b,f,g), possibly reflecting the presence of plastids that are lost in later stges of pollen development. Differences between CMS-S and N mito-types were apparent following the microspore mitosis but before significant starch accumulation. At this young pollen (YP) stage, the two nuclei of N-cytoplasm pollen were condensed (Fig. 1d), whereas those of CMS-S pollen were dispersed, with chromatin fragments at the periphery of the nuclear envelope (Fig. 1i,k). A single condensed chromatin mass was observed in collapsed CMS-S pollen (CP) (Fig. 1j). In contrast, N-cytoplasm pollen development proceeded by division of the generative nucleus to the tri-cellular, mature pollen (MP) stage (Fig. 1e).

**Fig. 2:**
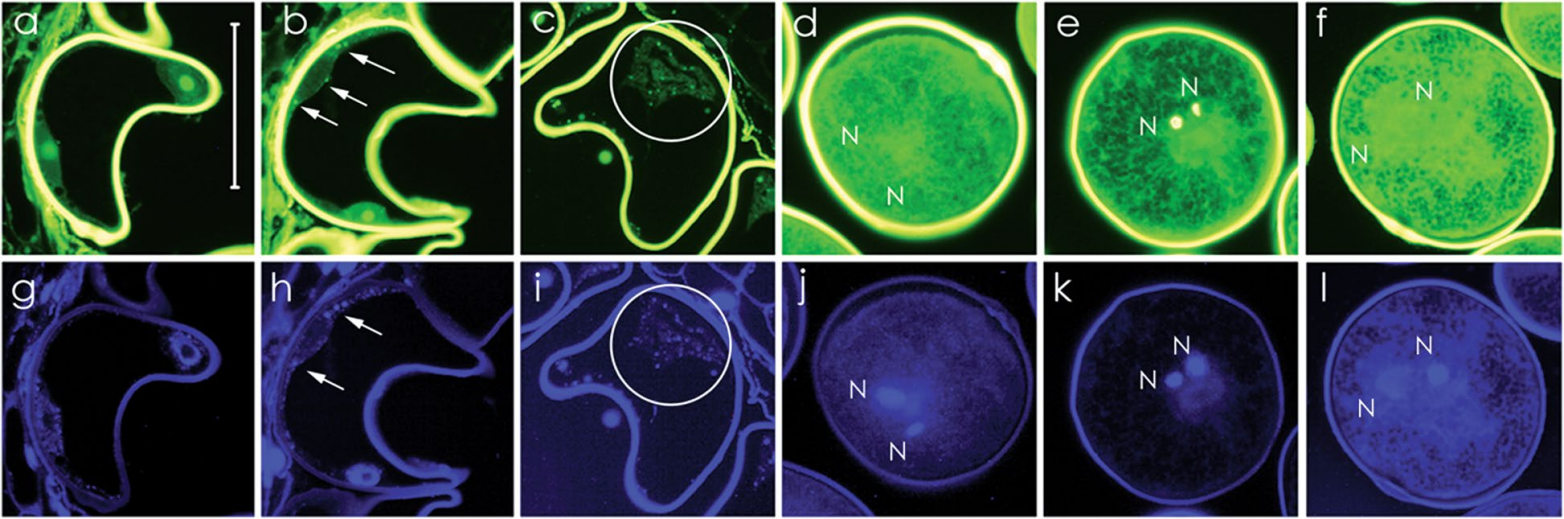
Terminal dUTP Nick End Labeling (TUNEL) assay and DAPI staining of normal (N) and CMS-S pollen. **a-l** Tissue sections of developing maize pollen dual stained with **a-f** TUNEL and **g-l** DAPI. **a**, **g** TUNEL-negative nuclei in N-cytoplasm young pollen (YP). **b**, **h** TUNEL-positive staining in the nuclei of CMS-S YP. **c**, **i** Fully collapsed CMS-S pollen (CP) showing condensed cytoplasm with punctate, TUNEL-positive bodies. **d**, **j** Starch-filling N-cytoplasm mature pollen (MP) showing no TUNEL staining. **e**, **k** TUNEL positive staining of MP pre-treated with DNase. **f**, **l** Negative control for e, k**.** Arrows, TUNEL-positive nuclear regions; circles, condensed, TUNEL-positive cytosol; n, Nuclei. Bars = 50 µm

The morphology of CMS-S CP pollen (Fig. 1) [31], and the involvement of mitochondria in both CMS of plants and apoptotic PCD of animals, pointed towards mitochondria-signaled PCD as the basis for the CMS-S pollen collapse. To test this hypothesis, terminal dUTP nick end labeling (TUNEL) assays were performed to examine BCP of CMS-S and N-cytoplasm mito-types for the presence of DNA breaks and nuclear fragmentation (Fig. 2), features of apoptotic PCD in animals [13, 33, 36]. Nuclei of the N-cytoplasm YP pollen were TUNEL-negative (Fig. 2a) whereas the nuclei of CMS-S YP showed bright green, punctate, TUNEL-positive staining (Fig. 2b). CMS-S CP showed TUNEL-positive packets throughout a disorganized cytoplasm (Fig. 2c). TUNEL assays of N-cytoplasm MP were negative (Fig. 2d,f) unless pollen was pre-treated with DNase (Fig. 2e). DAPI counter-stained DNA was observed in conjunction with TUNEL positive signals when they were present (Fig. 2h,i,k).

### Mitochondrial membrane potential, morphology and distribution during CMS-S and N-cytoplasm pollen development

**Fig. 3:**
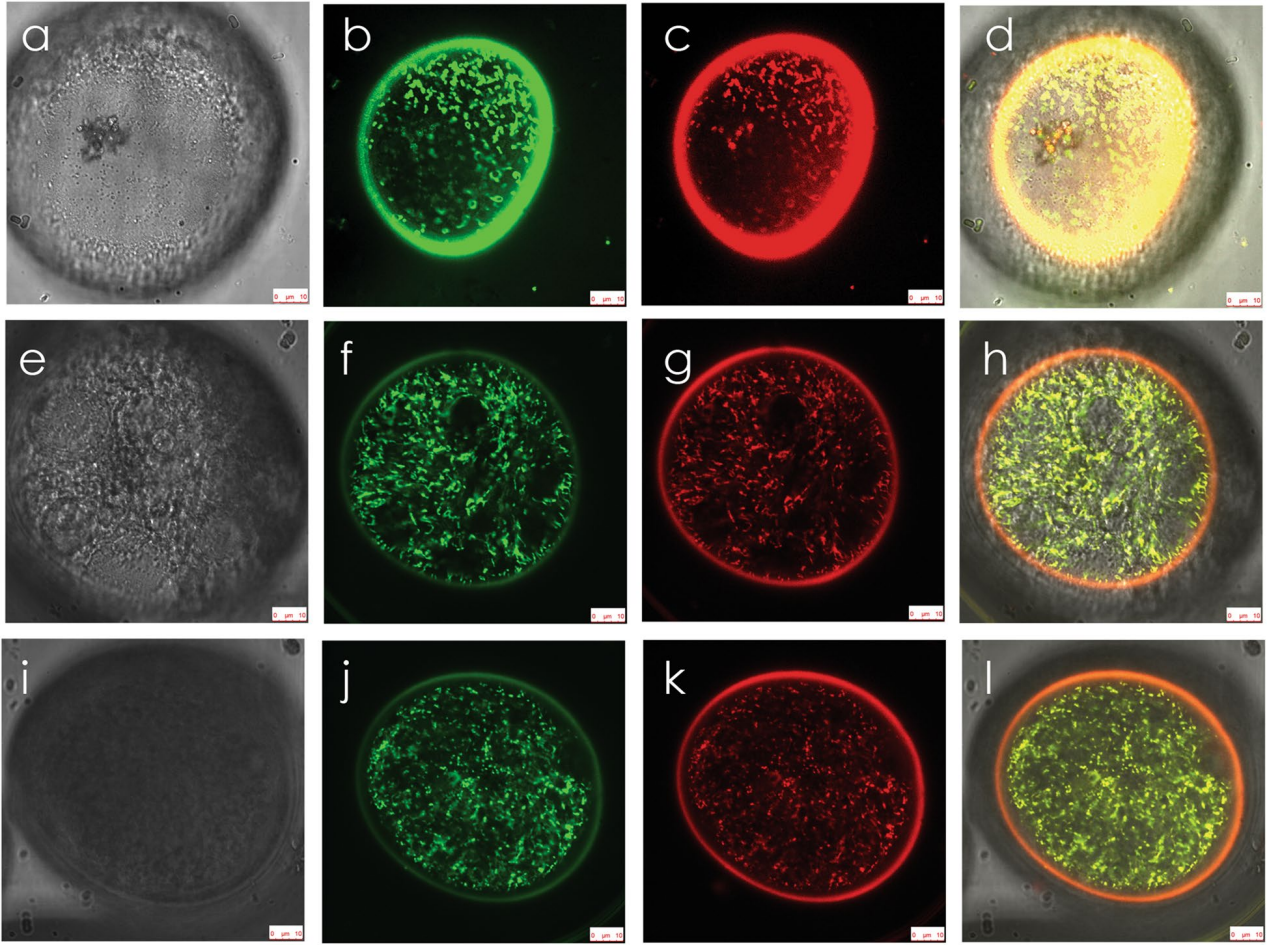
Mitochondrial targeting of green fluorescent protein (GFP) and mitochondrial membrane potential in normal (N) cytoplasm maize pollen development. **a-l** Scanning laser confocal images of **a-d** young, bi-cellular pollen (YP); **e–h** starch filling pollen (SFP); and **i-l** mature pollen (MP)**.** For each developmental stage, multiple images of the same pollen grain are shown. **a**, **e**, **i** Bright field digital interference contrast (DIC). **b**, **f**, **j** Pollen-expressed, mitochondria-targeted GFP. **c**, **g**, **k** Mitochondrial staining with the potentiometric dye JC-1. **d**, **h**, **l** Merged GFP and JC-1 images. Bars = 10 µm

**Fig. 4:**
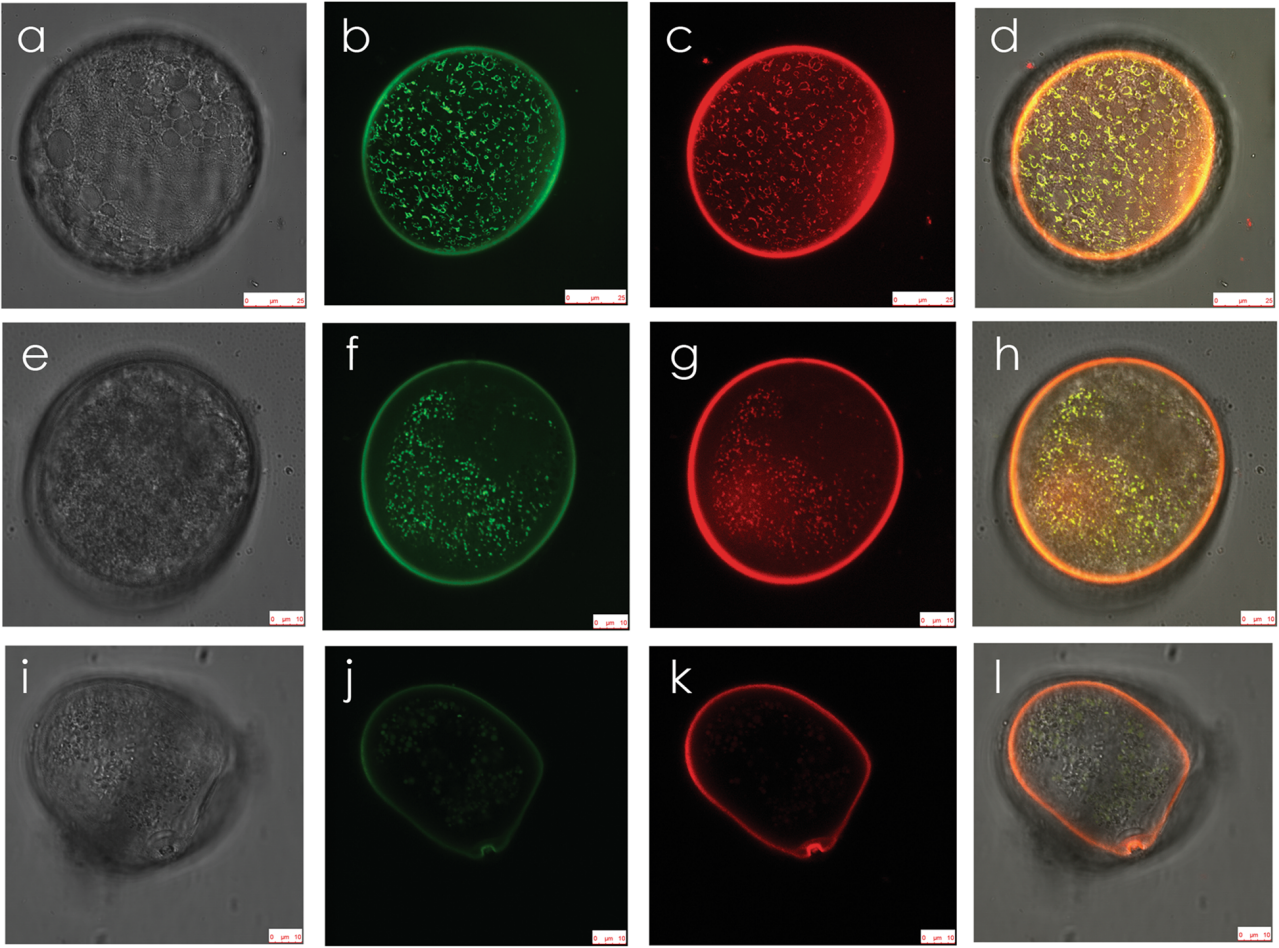
Mitochondrial targeting of green fluorescent protein (GFP) and loss of mitochondrial membrane potential in CMS-S maize pollen development. **a-l** Scanning laser confocal images of **a-d** young, bi-cellular pollen (YP); **e–h** pre-collapse, starch-filling pollen (PC); and **i-l** collapsed pollen (CP)**.** For each developmental stage, multiple images of the same pollen grain are shown. **a**, **e**, **i** Bright field digital interference contrast (DIC). **b**, **f**, **j** Pollen-expressed, mitochondria-targeted GFP. **c**, **g**, **k** Mitochondrial staining with the potentiometric dye JC-1. **d**, **h**, **l** Merged GFP and JC-1 images. Bars = 25 µm for **a-d**; 10 µm for **e-l**

Use of the maize *ZM13* promoter [37] and the tandemly duplicated *Neurospora crassa* ATP synthase subunit 9 (ATP9) mitochondrial targeting leader peptide [38] allowed for mitochondrial targeting of a green fluorescent protein (GFP) in maize pollen from the YP stage forward. This was demonstrated by laser-confocal scanning microscopy in both N (Fig. 3) and CMS-S (Fig. 4) mito-types. Bright field microscopy demonstrated similar morphology of N-cytoplasm and CMS-S YP (Fig. 3a, Fig. 4a, respectively). YP of both mito-types contained abundant GFP-labeled organelles, many of which were ring-shaped. These GFP signals co-localized with the red signals generated by the mitochondrial potentiometric dye JC-1 (Fig. 3b-d, Fig. 4b-d). Both N-cytoplasm and CMS-S YP therefore contained numerous mitochondria capable of generating a membrane potential and importing GFP. As the development of N-cytoplasm pollen advanced through the starch filling pollen (SFP) and MP stages, mitochondrial GFP and JC-1 signals remained robust (Fig. 3e-l). Punctate GFP and JC-1 signals continued to be detected in slightly advanced, pre-collapse (PC) CMS-S pollen (Fig. 4f-h), but were significantly diminished in CP samples (Fig. 4j-l).

**Fig. 5:**
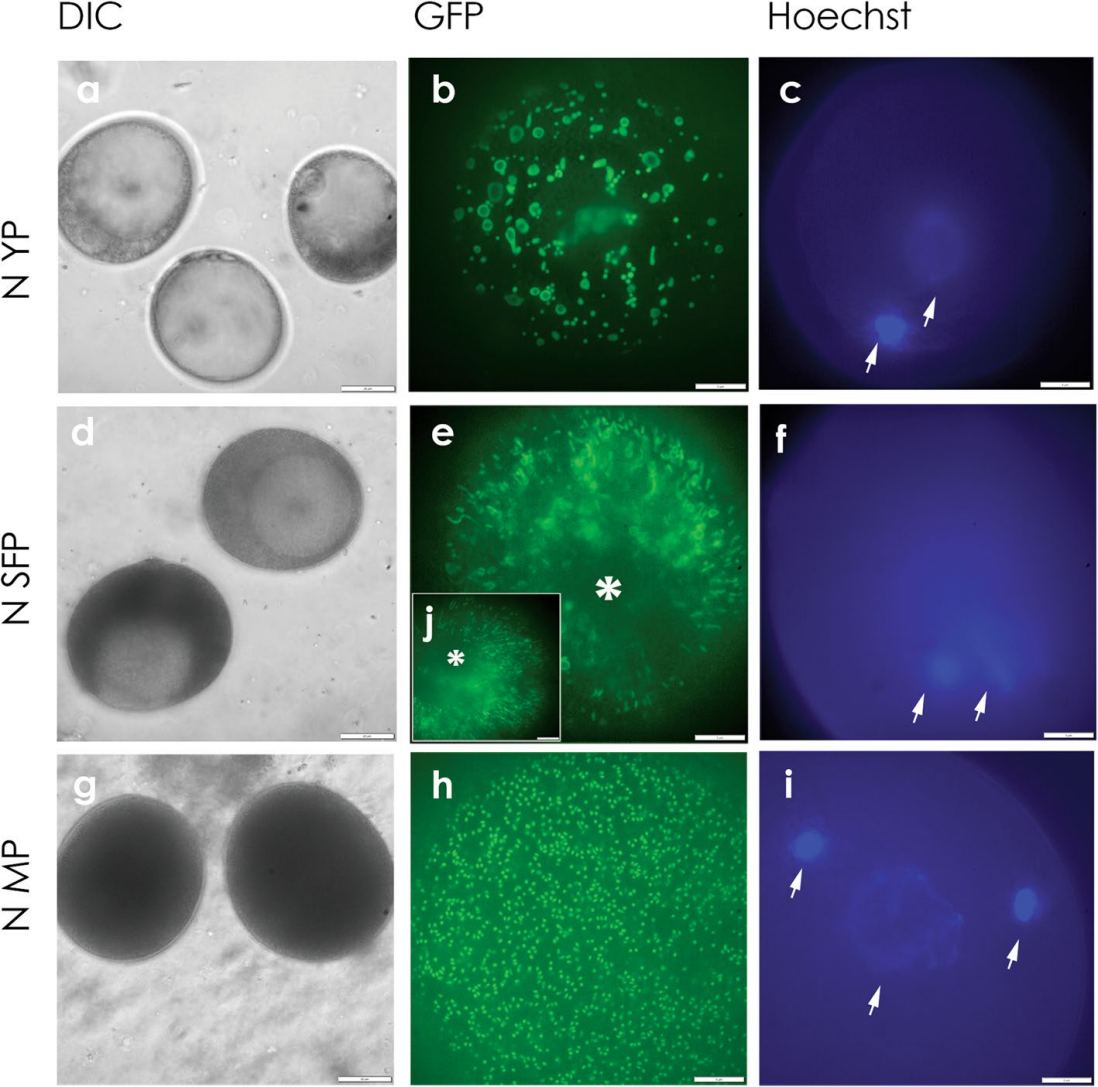
Mitochondrial morphology changes in normal (N) cytoplasm maize pollen development. **a-j** Spinning disc confocal micrographs of **a-c** young, bi-cellular pollen (YP); **d-f** starch filling pollen (SFP); and **g-i** mature pollen (MP). For each developmental stage, images of different pollen grains collected from the same anther are shown; **a**, **d**, **g** Bright field digital interference contrast (DIC). **b**, **e**, **h**, **j** Mitochondria-targeted green fluorescent protein **(**GFP). **c**, **f**, **i** Hoechst nuclear staining. **j** inset showing a transition stage between **e** and **h**. *, germination pore; arrows, Hoechst-stained nuclei. Bars = 20 µm for **a**, **d**, **g**; 5 µm for **b**, **c**, **e**, **f**, **h**, **i**, **j**

Pollen-expressed, mitochondria-targeted GFP also revealed striking changes in mitochondrial morphology during N-cytoplasm pollen development (Fig. 5). Viewed by spinning disk confocal microscopy, mitochondria of bi-cellular YP were fused, many into ring-shaped structures (Fig. 5b,c). These transitioned to fused linear arrays in SFP (Fig. 5e,f) and then to uniformly dispersed punctate organelles in MP (Fig. 5h,i). With this technique, which lacks the resolution of laser confocal microscopy, GFP labeled, CMS-S pollen mitochondria were largely obscured by diffuse, untargeted GFP at both YP and CP stages (Additional File 1, Supplemental Fig. 1).

**Fig. 6:**
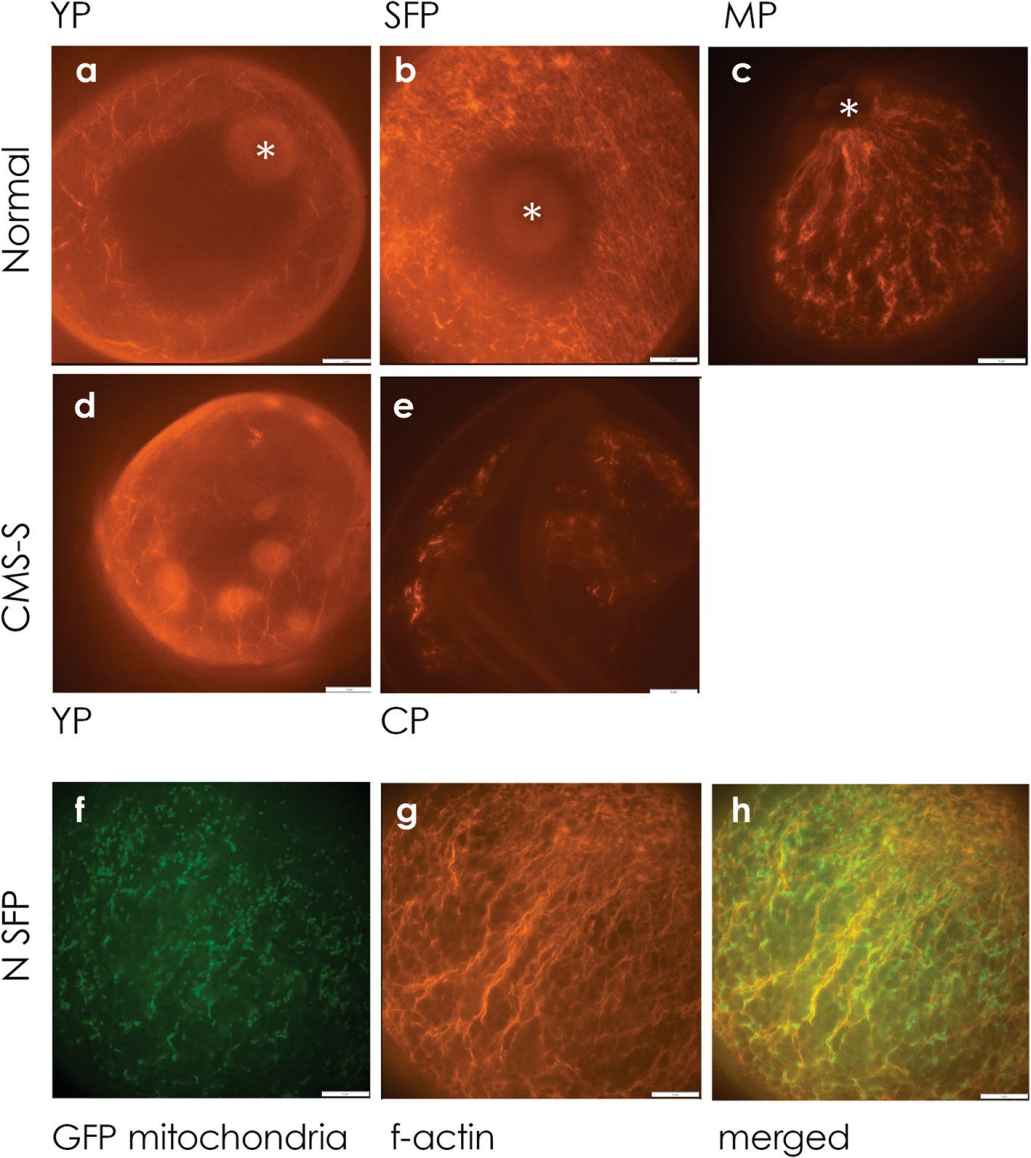
Actin filament organization and co-localization of mitochondria and actin filaments in maize pollen development. Spinning disc confocal micrographs of Phalloidin-iFluor 532 conjugate stained **a-c** normal (N) cytoplasm and **d-e** CMS-S pollen. **a**, **d** Lose actin network at the young pollen (YP) stage. **b**, **c** Organized, parallel actin arrays in N-cytoplasm starch-filling pollen (SFP) and mature pollen (MP), respectively. Parallel filaments run from the germination pore to the opposite side of the MP grain. **e** Disorganized, short actin filaments in CMS-S collapsed pollen (CP). **f–h** Spinning disc confocal micrographs of N-cytoplasm SFP. **f** Mitochondria-targeted green fluorescent protein (GFP) and **g** Phalloidin-iFluor 532 conjugate stained actin filaments (f-actin) of the same pollen grain. **h** Merged f and g images showing co-alignment of mitochondria with actin filaments. *, germination pore. Bars = 5 µm

The linear mitochondrial arrays in N-cytoplasm SFP suggested alignment with the cytoskeleton. Phalloidin-iFluor 532 staining, in combination with spinning disk confocal microscopy, revealed dispersed actin in both CMS-S and N-cytoplasm YP (Fig. 6a,d). Dispersed actin transitioned to organized, parallel actin arrays in N-cytoplasm SFP. At this stage the actin filaments were observed in multiple orientations with respect to the pollen germination pore (Fig. 6b), and the mitochondrial GFP signals largely co-localized with the actin filaments (Fig. 6f-h). In N-cytoplasm MP, the actin filaments took on a distinct orientation with respect to the germination pore (Fig. 6c), whereas mitochondria had become punctate and dispersed (Fig. 5h). CMS-S CP was characterized by disorganized actin clumps and short actin filaments (Fig. 6e).

### Mitochondrial protein composition changes through the course of pollen development

**Fig. 7:**
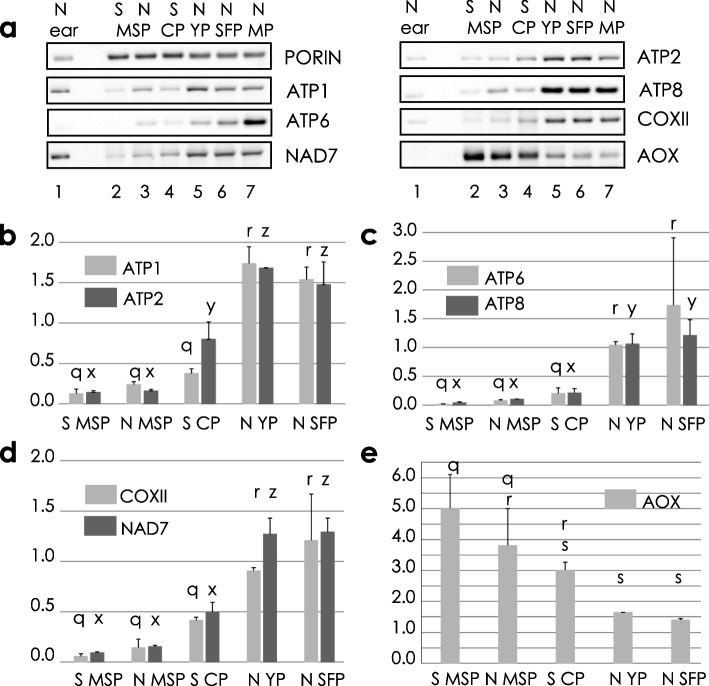
Developmentally regulated mitochondrial biogenesis in maize microspores and pollen. **a** Immunodetection of mitochondrial proteins in developing maize microspores and pollen. Proteins (ATP1, ATP2, ATP 6, ATP8, mitochondrial ATP synthase subunits 1, 2, 6 and 8, respectively; COXII, cytochrome oxidase subunit 2; NAD7, NADH dehydrogenase subunit 7; AOX, alternative oxidase) and the PORIN loading control were immunodetected following denaturing gel electrophoresis. Cropped blot images are shown boxed, and full length blots are presented in Additional File 2, Supplementary Fig. 2. Protein samples contained: Lane 1, 1 ug of protein extracted from normal (N) cytoplasm immature ear mitochondrial pellets; lanes 2–7, 10 ug of total protein extracted from N or CMS-S (S) microspore (MSP), or pollen stages: collapsed pollen (CP), young pollen (YP), starch filling pollen (SFP), mature pollen (MP). **b-e** Accumulation of mitochondrial proteins from CMS-S and N-cytoplasm maize microspores and developing pollen as compared to mature, N-cytoplasm pollen (set equal to 1). Error bars correspond to the standard deviation for three biological replicates. **q**,**r**,**s** different letters distinguish samples that differ in the relative abundance of ATP1, COXII, ATP6 or AOX at *p* ≤ 0.05 by a post-ANOVA Tukey’s HSD test. **x**,**y**,**z** different letters distinguish samples that differ in the relative abundance of ATP2, ATP8 or NAD7 at *p* ≤ 0.05 by the same test

The collapse of CMS-S pollen following the microspore mitosis pointed to a developmental trigger for this PCD event. Immunoblotting studies (Fig. 7a, Additional file 2, Supplemental Fig. 2) were conducted to investigate mitochondrial protein features of CMS-S and N-cytoplasm pollen development, collected and staged by sucrose density gradients as described in Methods. Because the numbers of mitochondria vary between stages of maize pollen development [39], the nuclear-encoded, mitochondrial outer membrane protein PORIN was used as a reference standard against which the abundance of mitochondrial respiratory proteins was compared at each developmental stage, with between-stage comparisons expressed relative to N-cytoplasm MP (Fig. 7b-e). CMS-S and N-cytoplasm MSP had similar mitochondrial protein accumulation patterns, with low abundance of cytochrome respiratory pathway components. The abundance of MSP respiratory complex I (NAD7), IV (COXII) and V (ATP1, ATP2, ATP6 and ATP8) subunits did not differ significantly between CMS-S and N mito-types. The abundance of these proteins was, however, significantly lower in MSPs compared to pollen stages. In N cytoplasm, ANOVA demonstrated that significantly increased accumulation of respiratory complex subunits accompanied the transition from MSP to the YP stage, ranging from six fold for COXII to 13- fold for ATP6 (Fig. 7b-d). Although not all were significant in the ANOVA analysis, increased abundance of respiratory proteins was apparent in comparisons between CMS-S MSP and CP and was significant for ATP1, ATP2, ATP6, COXII, and NAD7 at p ≤ 0.05 in a one-tailed T test. Another striking protein feature of the MSP stage for both mito-types was the high abundance of the nuclear-encoded AOX, believed to protect against the production of reactive oxygen species (ROS) by the respiratory chain. In both CMS-S and N-cytoplasm pollen development, the transition from MSP to BCP was accompanied by a significant decrease in AOX (Fig. 7e). CMS-S pollen collapse therefore occurs concomitant with a developmentally programmed shift in the mitochondrial protein profile when respiratory membrane protein complexes are being accumulated and AOX protection is declining.

### Robust mitochondrial transcript editing in maize microspores

Multiple plant mitochondrial gene expression control points [40] potentially contribute to the low abundance of mitochondrial gene products in developing MSPs. These include C-to-U RNA transcript editing required for correct amino acid coding, observed as C-to-T changes in cDNA sequences (reviewed in [41]). RNA blot hybridizations have shown that mitochondrial ribosomal RNAs and transcripts encoding respiratory proteins accumulate in developing CMS-S and N-cytoplasm MSPs [35]. Moreover, sequencing of cDNA clones confirmed that *atp9* transcripts are edited at the MSP stage in both mito-types [42]. Here, these findings were extended to *atp6* and *atp8* transcripts. RNA blot hybridizations show similar mitochondrial transcript patterns between CMS-S and N-cytoplasm MSPs and immature ears or N SFP, although the relative abundance of the different transcript forms showed developmental differences (Additional file 3, Supplemental Fig. 3a). Direct sequencing of *atp6* and *atp8* cDNAs amplified from mitochondrial RNAs of immature ears established the RNA editing patterns for these transcripts (Additional file 4, Supplemental tables 1,2). Examination of cDNA sequence traces confirmed robust editing of *atp6* and *atp8* transcripts at the MSP stage in both mito-types. In the case of *atp6*, all 15 codon-changing sites were fully edited in CMS-S and N-cytoplasm ears and MSPs. Representative traces are shown in Additional File 3, Supplemental Fig. 3b. The *atp8* transcripts contained three codon-altering edits. These sites were fully edited in immature ear transcripts. While there were traces of partial editing at codon 20 in both CMS-S and N-cytoplasm MSPs, the edited codon always predominated. Codon 146 contained two editing sites. The CCA (P) codon was edited to either a CTA (L) or a TTA (L) codon, with partial editing at the first position in both ear and MSP samples. In S MSPs, there was evidence of partial editing at the second position, but again the edited sequence predominated in the trace (Additional file 3, Supplemental Fig. 3c).

## Discussion

### CMS-S pollen collapse is a programmed cell death event

CMS is conditioned by a variety of mitochondrial open reading frames and a corresponding diversity of cellular features, ranging from the homeotic transformation of the pollen-producing stamens into carpels or petals to death of the tapetum, developing microspores, or pollen [1, 3, 4, 6, 16]. In the homeotic types, mitochondrial retrograde signaling events impact the expression of nuclear floral organ identity genes [2]. In light of mitochondria-signaled PCD in animal systems [21, 43–46] and mitochondrial events associated with PCD in plants [15, 17, 23, 24, 28, 47], mitochondrial cell death signaling is another possible route to CMS.

CMS-S pollen collapse presents a plant cell death cytology very like that of animal cells undergoing mitochondria-signaled apoptosis. Collapsed CMS-S pollen reveals condensed cytoplasm containing membrane-bound packets and membranous whorls [31] similar to those described for apoptotic animal cells PCD [21, 33]. The DNA breaks and subsequent nuclear fragmentation observed in CMS-S pollen (Fig. 1, Fig. 2) also resemble key elements of apoptosis in animals (reviewed in [48, 49]). DNA features of CMS-S pollen are similar to those of PET1-CMS sunflower, but the two systems differ with respect to the developmental timing and tissues affected. In the sunflower system, DNA cleavage is first observed in the diploid tapetal cells of the anther [12]. PET1-CMS is therefore a sporophytic system, whereas in CMS-S maize all events take place in the haploid gametophyte. PCD processes are important to plant growth, development and defense [17–20]. While mitochondria feature as key signaling elements in both plant and animal PCD systems, downstream components show functional conservation but structural variation [27]. Given morphological similarities between CMS-S pollen collapse and canonical events of apoptotic PCD in animals, it will be of interest to identify the downstream components of this pollen collapse pathway.

### Mitochondrial morphology changes during CMS-S and N-cytoplasm pollen development

Transgenic maize plants, in which GFP is expressed from the maize *ZM13* promoter and targeted to the mitochondria by the *N. crassa* ATP9 mitochondrial targeting leader, revealed mitochondrial behavior from the YP stage forward. In both N-cytoplasm (Fig. 3a-d) and CMS-S (Fig. 4a-d) YP, laser confocal microscopy revealed GFP effectively targeted to the mitochondria, confirmed with overlapping red JC-1 mitochondrial membrane potential signals. While mitochondrial membrane potential persisted throughout the course of N-cytoplasm pollen maturation (Fig. 3e-l), absence of JC-1 signals associated loss of mitochondrial membrane potential with CMS-S pollen collapse (Fig. 4e-l). Although we were unable to determine the exact timing of this event with respect to pollen collapse, loss of mitochondrial membrane potential is frequently an early feature of plant PCD [50].

A striking evolution of mitochondrial morphology was observed through the course of N-cytoplasm pollen development, but was not fully elaborated in CMS-S pollen. These morphological transitions likely reflect the differing roles and functions of mitochondria through the course of pollen development. Mitochondria-targeted GFP, observed with either laser confocal or spinning disk confocal microscopy, revealed fused mitochondria, including ring-shaped structures, in N-cytoplasm YP (Fig. 3b; Fig. 5b). In CMS-S YP this morphology was only apparent with laser confocal microscopy (Fig. 4b) as the laser could penetrate deeper into the pollen making it the more sensitive method. Under spinning disk confocal observation (Additional File 1, Supplemental Fig. 1), all stages of the CMS-S pollen showed a diffuse and sometimes mottled GFP signal, indicating that not all of the GFP was being targeted to or incorporated into the mitochondria [51]. Plant mitochondrial genomes exist as complex, multipartite structures that are not uniformly distributed among the organelles [6, 52]. Mitochondrial fusion unites mitochondrial genes and gene products needed for biogenesis of functional mitochondria [53, 54]. This is observed in de-differentiating mesophyll protoplasts [55], shoot apical meristems [56], zygotes [57] and germinating embryos [58]. The mitochondrial fusion observed in maize YP potentially supports the accumulation of respiratory proteins observed during pollen maturation (Fig. 7).

In N-cytoplasm pollen development, fused mitochondria transitioned to long linear arrays at the SFP stage (Fig. 3f, Fig. 5e) and then to dispersed, punctate mitochondria in MP (Fig. 3i, Fig. 5h). This is similar to events observed in tobacco mesophyll protoplasts, where mitochondrial fission transitions fused organelles to punctate forms prior to cell division [59]. The transition to dispersed, punctate form is needed for proper pollen function. The Arabidopsis GTPase MIRO1 is required for mitochondrial fission. Loss of MIRO1 function impairs growth and germination of haploid pollen due to the accumulation of enlarged mitochondria that disrupt mitochondrial streaming in the pollen tubes [60]. The enlarged mitochondria of MIRO1 mutant pollen are similar to those seen in maize YP (Fig. 3b, Fig. 4b, Fig. 5b).

Actin filament organization was associated with the mitochondrial patterning events observed in developing maize pollen (Fig. 6). The actin cytoskeleton is essential for uniform distribution and inheritance of organelles during tobacco protoplast division [59], and plant mitochondria array and move on microtubule-positioned actin filaments in a diversity of plant cell types [61–64]. In N-cytoplasm maize pollen, loose actin arrays observed in YP (Fig. 6a) transitioned to strong linear arrays (Fig. 6b) that co-localized with linear arrays of GFP-labeled mitochondria (Fig. 6f-h). Filamentous actin (F-actin) and tubulin align in a polar orientation transverse to the long axis of a cell during elongation [61]. In late pollen development, this actin organization is necessary for pollen germination and pollen tube growth [65, 66]. In maize SFP, actin arrays were initially seen both parallel and transverse to the axis defined as germination pore to opposite pole, and did not form in the region of the germination pore (Fig. 6b). In mature pollen, actin arrays became strongly oriented parallel to this axis and extended up to the germination pore in preparation for pollen tube growth [65, 67].

The CMS-S pollen mitochondria do not follow the ring – linear array – punctate morphology changes observed in N-cytoplasm pollen development, but instead take on a more punctate distribution in PC pollen (Fig. 4f). This is consistent with the disorganized short actin filaments seen in CP (Fig. 6e). Possibly the CMS-S mitochondria are unable to deliver the requisite amount of ATP necessary to initiate or maintain actin filament formation [67, 68]. This might contribute to, or be a consequence of, PCD initiation. Additionally, the inability to form actin filaments may lead to a lack of structural support and the collapsed pollen grain phenotype characteristic of CMS-S pollen upon death.

### Developmentally regulated mitochondrial biogenesis in maize pollen

Developmentally programmed changes in mitochondrial protein composition suggest a model in which these changes influence the timing of PCD in CMS-S pollen shortly into the BCP stage. At the MSP stage, cytochrome pathway and ATP synthase subunits are of very low abundance (Fig. 7a-d), whereas AOX is highly abundant (Fig. 7a,e). Mitochondria increase in numbers through maize MSP development but have a reduced internal membrane structure [69] that is consistent with the lack of respiratory proteins. MSP mitochondrial protein features might stem from the hypoxic state of the developing anther [20]. Maize germ cell initials are enriched in transcripts of genes supporting glycolysis and transcripts of *aox3* [70]. The hypoxic environment that specifies meiotic cell fate is hypothesized to also minimize ROS-mediated DNA damage during gametogenesis [71]. The transition from MSP to BCP is characterized by increased abundance of the cytochrome respiratory pathway and ATP synthase protein complex subunits and declining abundance of AOX (Fig. 7a-e). Activation of the cytochrome pathway in BCP would increase the potential for ROS production [72–74], while AOX, which transfers electrons from ubiquinone to oxygen by-passing respiratory complexes III and IV, protects against the generation of mitochondria-generated ROS [75–77]. ROS are potent cell death signaling molecules of plants in a variety of contexts [74, 78], and ROS have been suggested as a unifying component of the diverse plant CMS pathways [16]. The up regulation of oxidative stress induced genes and the down regulation of AOX-encoding genes are associated with the female phenotype in two different CMS systems of *Silene vulgaris* [79]. In CMS-S maize pollen, the accumulation of cytochrome pathway proteins and declining abundance of AOX coincides with pollen collapse at the BCP stage. In animal systems, mitochondrial ATP synthase subunits can function as components of a mitochondrial permeability transition pore associated with the release of apoptosis promoting proteins into the cytosol [80, 81], although alternative channels also function in this way [82]. It remains to be investigated whether PCD promoting molecules are released from CMS-S maize mitochondria and whether accumulating ATP synthase subunits facilitate such an event.

While transcripts of the CMS-S mitochondrial locus encode at least three potential open reading frames [42, 83], a *ubiquitin1* promoter-driven transgene encoding a mitochondria-targeted version of the ORF355 component effectively conditions maize pollen collapse [84]. Transcripts of mitochondria-encoded *orf355* [83] and the *orf355* transgene [84] are abundant in MSPs, but pollen collapse occurs at the bi-cellular stage in both natural and transgenic systems. Multiple processes of plant mitochondrial gene expression underlie the final accumulation of respiratory protein complexes [40]. C-to-U RNA transcript editing is required for correct amino acid coding of many mitochondrial gene products [41]. While there is no evidence for editing of the mitochondrial *orf355* sequences, the co-transcribed upstream *orf77* is edited at the MSP stage [42] and loss of editing is associated with fertility restoration by the nuclear *Rf3* gene [85]. Maize MSPs also exhibit accumulation and editing of mitochondrial transcripts encoding ATP6 and ATP8, proteins that do not become abundant until the BCP stage (Additional File 3, Supplemental Fig. 3; Fig. 7). It is not yet known whether the translational or post-translational controls that limit accumulation of these respiratory proteins also regulate accumulation of mitochondria-encoded ORF355 such that endogenous ORF355 accumulates at only at the BCP stage, or whether ORF355 accumulates in CMS-S MSP but cannot effect pollen collapse without concomitant mitochondrial events. CMS-S maize is unique in that it can be reversed by numerous nuclear *restorer-of-fertility* mutations, many of which compromise pollen mitochondrial gene expression [86]. The effects of these mutations on both the accumulation of mitochondria-encoded ORF355 and the phenotype of transgenic plants expressing mitochondria-targeted ORF355 could be useful in dissecting the relative independence of this protein as an executor of PCD in developing maize pollen.

## Conclusions

Our findings demonstrate dynamic changes in mitochondrial morphology and significant regulation of mitochondrial biogenesis through the course of N-cytoplasm maize pollen development. The accumulation of cytochrome respiratory pathway components and declining abundance of AOX are concomitant with apoptosis-like PCD in the CMS-S mito-type. Maize pollen development, the CMS-S mito-type, and its associated restorer mutations can be further probed for deeper understanding of mitochondrial biogenesis and its possible contributions to cell death signaling in plants.

## Methods

### Plant materials

Pollen development was compared between greenhouse-grown, CMS-S and N-cytoplasm versions of Mo17 inbred maize. For cytological and molecular studies, samples containing pre-MSP and MSP stages were prepared from pre-emergent tassels, while anthers containing BCP to MP stages were recovered from post-emergent tassels. For investigations of pollen mitochondrial morphology, transgenic maize lines were developed to express the redox sensitive S65T enhanced green fluorescent protein (roGFP2) [87] fused to the ATP9 double leader (ATP9DL) sequence that targets nuclear-encoded ATP9 to the mitochondria in *N. crassa* [38]. The *ATP9DL-roGFP* sequence was synthesized by Genscript USA coupled to the maize pollen-specific *ZM13* promoter, which initiates expression shortly after microspore mitosis and continues through late pollen development [37]. The Construct was cloned into an *Eco*RI-*Sac*I digested pTF102 vector [88]. The pTF102 clone was introduced into the maize Hi-II genotype [89] through *Agrobacterium*-mediated transformation at the Iowa State University Plant Transformation Facility (ISUPTF). Eight independent Hi-II T0 transformants were pollinated with the N-cytoplasm B73 inbred line (B73-N) at the ISUPTF. T0/B73-N seeds were grown at the University of FL and the plants were pollinated with B73-N to create T0//B73-N. T0/B73 plants were also used to pollinate CMS-S cytoplasm B73 plants (B73-S) creating paired N and S cytoplasm versions as back-cross 1 (BC1) in the B73 genetic background. To better match genetic materials used for non-transgenic cytology and molecular studies, three independent events (T0//B73-N) were subsequently crossed as pollen parents onto the Mo17-S and Mo17-N inbred backgrounds. Progeny were grown and subsequently pollinated with Mo17-N to create BC1 Mo17-S and Mo17-N pairs for these events.

### Fluorescence microscopy of DAPI stained pollen

Fresh pollen and microspores were stained with 600 nM 4',6' diamino-2-phenylindoleּHCl (DAPI) nuclear stain in McIlvaine’s buffer (20 mM citric acid, 40 mMNa_2_HPO_4_, pH 7.0). Pollen and microspores were teased out of the anthers into 15 μl of McIlvaine’s buffer on a glass slide. A 50 μl volume of DAPI stain was then added to the sample. Samples were then transferred from slides to microfuge tubes by pipet, and 2 μl (or 1 μl for microspore mother cells and tetrads) of acidified Orcein red (2% Orcein red in 45% acetic acid) was added. The sample was gently mixed by pipette and incubated for 20 min on a shaker in the dark at room temperature. The samples were then spun at 500 × g for 2 min. The supernatants were removed by pipet. Samples were rinsed in 50 μl McIlvaine’s buffer for 5 min, transferred to slides and protected with a cover slip. DAPI samples were viewed with a Leica microscope equipped with a 100 W mercury lamp for fluorescent illumination. Samples were excited at 350/50 × with emission at 460/50 m via the 31000v2 DAPI filter set (Chroma Technologies, USA). Micrographs were captured with an Insight CCD digital color camera using SPOT software version 3.2.4. (Diagnostic Instruments Inc., USA).

### TUNEL assays

Anthers were cut in thirds in a drop of fixative on a glass slide and then placed in 500 μl tubes filled with fixative (4% formaldehyde and 0.25% glutaraldehyde in 100 mM cacodylate buffer, pH 7.0) and incubated for 24 h at 4 °C. The samples were then rinsed in phosphate buffered saline (PBS) containing 137 mM NaCl, 2.7 mM KCl, 10 mM Na_2_HPO_4_, 1.8 mM KH_2_PO_4_ for 1 h on ice and subsequently dehydrated in a 10% to 100% ice cold ethanol series carried out in 10% increments for 20 min per step. The 100% step was performed twice. The dehydrated samples were infiltrated with Technovit 8100 resin (Electron Microscopy Sciences, USA) in a 1:1 resin:ethanol mix for 6 h followed by 100% resin overnight. The catalyst was added and samples were then embedded in the 500 μl tubes. The sample tips were cut off with a jeweler’s saw then mounted on aluminum mounting stubs and sectioned on a Sorvall Porter-Blum MT2-B ultra microtome. The sections were cut to 500 nm thick.

Terminal deoxynucleotidyl transferase (dUTP) Nick End Label (TUNEL) assays (Roche, Switzerland) were performed according to manufacturer’s instructions. The samples were washed twice in PBS for 5 min per wash. Proteinase K was added to a final concentration of 20 µg/ml. Samples were incubated for 20 min at 37 °C in a humid chamber, and rinsed with PBS. At this point, positive controls were treated with 3,000 units of DNase I for 10 min at room temperature and rinsed with PBS. All samples were treated with the TUNEL reaction mix for 1 h at 37 °C in a humid chamber in the dark. Negative controls were incubated in the absence of the fluorescent labeled dUTP. The remaining steps were performed in low light. Samples were rinsed in PBS and counterstained with 600 nM DAPI in Fluoromount (Life Technologies, USA). TUNEL samples, also viewed with a Leica microscope equipped with a 100 W mercury lamp, were excited at 470/40 × with emission at 500lp via a 41,018 Endow GFP/EGFP long pass filter (Chroma Technologies, USA).

### Pollen mitochondrial morphology studies

Laser confocal microscopy was used to confirm pollen expression of the *ZM13-ATP9DL-S65T-gfp* construct and mitochondrial targeting of the GFP protein. Three stages of pollen development were examined for two independent transgenic events for B73-N BC1 and B73-S BC1. The developmental stages were determined by viewing pollen under bright field microscopy and selected based on starch levels. YP containing very little starch, SFP roughly 50%-75% filled with starch, and 100% starch filled MP at the point of anther dehiscence and were examined. To ensure the stages of pollen were consistent, flowers were pulled from the same area of the tassel and dissected so that the upper and lower florets were separated. The anthers from the upper and lower florets were minced in tap water and examined separately under a microscope to determine which stage best suited the criteria needed. YP was staged from flowers harvested when the tassel was partially emerged, SFP from the tassel when it was almost fully emerged and MP from tassels at the start of dehiscence.

To confirm GFP targeting to the mitochondria, the DePsipher Mitochondrial Potential Assay kit was used (Trevigen Inc., USA). JC-1 was prepared according to the DePsipher assay directions with a final working solution of 5 µg/ml. Pollen was removed from the anthers as described above, incubated in the dark at 28 °C with shaking for 45 min, spun at 1,000 xg for 4 min, rinsed with the buffer provided and viewed immediately. Scanning laser confocal images of JC-1 stained, S65T-GFP-tagged mitochondria in both CMS-S and N-cytoplasm pollen were taken with a Leica TCS-SP5 microscope using argon 488 nm laser line, 63 × oil objective, and Leica Microsystems LAS AF 2.6.0.7266 software (Leica Microsystems Inc., Germany).

Spinning disk confocal microscopy (DSU) was used to investigate mitochondrial morphology changes through CMS-S and N-cytoplasm pollen development in three BC1 Mo17-S and Mo17-N paired transgenic events. Pollen freshly removed from anthers was left in tap water and viewed with an Olympus IX81 inverted DSU microscope using Cellsens Dimension v2.3 software (Olympus Corp., USA) and a Hamamatsu C11440 digital camera (Hamamatsu Corp., USA) with a 40 × dry lens for bright field images and a 150 × oil lens (NA 1.45). A Chroma 49,002 FITC filter (Chroma Technology Corp., USA) was used to examine the pollen. Nuclei were stained with Hoechst 33,342 (Life Technologies, USA) and visualized with a Chroma 49,000 DAPI filter. The pollen nuclei could not be efficiently stained with Hoechst while simultaneously examining the GFP-tagged mitochondrial morphology due to the staining procedure altering the appearance of the mitochondria. Therefore, the sample was split in half to view the mitochondria and nuclei separately.

Because Maize pollen is difficult to stain, a partial sporoplast procedure [90] was employed prior to Hoechst nuclear staining. Briefly, pollen was isolated as described above, placed in a 1.5 ml centrifuge tube, and spun for 4 min at 1,000 xg. The tap water was removed by pipette. One ml of hydration buffer containing 300 mM pentaerythritol, 3 mM 2-(N-morpholino)ethanesulfonic acid (MES), 9 mM CaCl_2_, pH 5.2 was added to the tube and the entire sample transferred to a scintillation vial. The sample was gently agitated for 10 min. Then 1 ml of 60% 4-methylmorpholine N-oxide in aqueous solution was added to the vial, and the pH adjusted to 7.5 with 50 µl 1 M Trizma base, pH 8.0. The sample was stirred with a glass pipette to remove any pollenkitt, collected by vacuum filtration, and rinsed with wash buffer (300 mM pentaerythritol, 3 mM MES, 4.5 mM CaCl_2_, pH 7.0). The pollen on the filter was rinsed into a scintillation vial with 1 ml of the wash buffer. For Hoechst staining the sample was transferred into a 1.5 ml centrifuge tube and spun for 4 min at 1,000 xg. The wash buffer was removed by pipet and, in dimmed light, 100 µl of Hoechst stain (2.5 µg/ml in wash buffer) was added. The sample was incubated in the dark with shaking at 28˚C for 30 min, collected by centrifugation as before and resuspended in wash buffer. Samples of 50 µl were placed on slides for immediate viewing.

### F-actin staining

Pollen was isolated as previously described. iFluor 532 actin stain (AAT Bioquest Inc.) was prepared as described by the manufacturer. Isolated pollen was fixed in 4% paraformaldehyde in PBS pH 7.0 for two h in the dark at room temperature with agitation and was then placed in the refrigerator overnight. The pollen was centrifuged for 4 min at 1000 xg. The fixative was removed and a PBS wash was added. The pollen was washed for 5 min with agitation and pelleted. The supernatant was removed and the wash was repeated for an additional 15 min. The supernatant was removed after another spin, and 200 µl iFluor 532 added. The pollen was incubated for 45 min in the dark at room temperature with agitation. The pollen was viewed with a DSU microscope, 150 × oil objective as previously described and a TRITC filter (Chroma 49,010), ET546(10x)/T560lpxr/ET585(40 m). All microscopy figures were prepared using Adobe Photoshop CS5 v12 × 32. GFP and actin DSU images were deconvolutions and/or projections of Z-stacks.

### Protein analysis

The procedures of Boutry and Briquet [91] were used to prepare crude mitochondrial pellets from immature ears of CMS-S or N-cytoplasm Mo17 inbred maize. Preparative-scale recovery of developing pollen was performed as described by Bedinger and Edgerton [34], except that separation of the stages was performed on step gradients in an HB-4 rotor spun at 8,000 xg for 20 min at 15 °C. Uni-nucleate MSPs were recovered from the buffer-50% sucrose interface. YP with little starch (collapsed in the case of CMS-S) was recovered from the 50%—70% sucrose interface, and SFP formed a pellet at the bottom of the 70% sucrose layer. MP was collected on filter paper as it was shed from the anthers. For all stages, samples of 0.1—0.25 g were transferred to 1.5 ml centrifuge tubes, frozen in an aluminum block equilibrated to -80 °C and stored at -80 °C.

Pollen protein accumulation was assayed by denaturing gel electrophoresis and immunoblotting. Total detergent soluble proteins were recovered from frozen microspore or pollen samples crushed in a mortar in the presence of 1X NuPAGE® LDS sample buffer (Thermo-Fisher Scientific Inc., USA) containing 50 mM dithiothreitol and 1.0 mM phenyl methyl sulfonyl fluoride but no tracking dye. A ratio of 200 ul buffer per 0.1 g of sample was used. The extract was transferred to a 1.5 ml centrifuge tube and incubated at 70 °C for 10 min with intermittent vortex mixing. Insoluble materials were pelleted by two centrifugations, each for 10 min at 12,000 xg. The final supernatants were recovered and stored at 10 °C. Aliquots of each extract were diluted 1/40 in sterile distilled water, and the Pierce™ Detergent Compatible Bradford Assay Kit (Thermo-Fisher Scientific Inc.) was used to determine protein concentrations of the diluted extracts. The original, undiluted extracts were then diluted in NuPAGE® sample buffer containing tracking dye to achieve a protein concentration of 1.0 µg/ml. Protein samples were fractionated by electrophoresis through pre-cast NuPAGE® gels, transferred to 0.45 micron nitrocellulose membranes and decorated with primary antibodies followed by horseradish peroxidase (HRP)-conjugated secondary antibodies. The primary antibodies used in this work are described in Additional File 5 Table S3. Mitochondria change in number during maize pollen development [39], and the nuclear-encoded, mitochondrial the outer membrane protein PORIN was used as a reference standard for comparison with mitochondrial respiratory complex subunits. Duplicate gels and blots were prepared for each of three independent sample sets. Each blot was first processed with the PORIN antibody. Blots were subsequently processed sequentially with additional antibodies recognizing mitochondrial proteins of different sizes so that blots did not have to be stripped between antibodies. Chemiluminescent signals were generated by incubating the blots in SuperSignal™ West Pico Plus chemiluminescent horseradish peroxidase substrate (Thermo Fisher Scientific Inc.). Blots were imaged in a ChemiDoc ARS + System (Bio-Rad Laboratories, USA). Images were captured and band volumes were determined with Image Lab™ software, version 6.1 (Bio-Rad Laboratories). The respiratory subunit/PORIN ratios of each sample were normalized to the ratio observed in the mature pollen sample on the same blot. The relative abundance of AOX, which was not effectively separated from PORIN, was calculated relative to the average abundance of PORIN determined from two replicate blots of the same sample series. For each protein of interest, the ratios from the three independent sample sets were averaged. Means and standard deviations for these ratios were calculated in Excel. Protein accumulations were compared through use of the one-way ANOVA with post-hoc Tukey honestly significant difference test calculator https://astatsa.com/OneWay_Anova_with_TukeyHSD/ accessed (8/12/2022).

### RNA analysis

Crude mitochondrial pellets were prepared from CMS-S and N-cytoplasm immature ears, MSPs and pollen by the procedure of described by Boutry and Briquet [91]. RNA was extracted in the presence of guanidine thiocyanate, denatured with glyoxal and fractionated by agarose gel electrophoresis as described by Wen and Chase [35]. Ribosomal RNAs were detected by ethidium bromide staining. RNAs were transferred to non-charged nylon membranes, hybridized to full-length coding sequence probes labeled with the BrightStar® BioDetect™ system (Thermo Fisher, USA) and detected by exposure to X-ray film. cDNAs were generated on mitochondrial RNA templates, amplified, and sequenced as described by Gallagher et al. [42]. Primers used in this work are described in Additional file 5, Supplemental Table 4. RNA editing sites were identified by comparing cDNA sequences to published maize mitochondrial genomic sequences (*Zea mays* strain NB mitochondrion, complete genome (NC_007982.1) and *Zea mays* subsp. mays genotype CMS-S mitochondrion, complete genome (DQ490951.2). cDNA sequences submitted to Genbank are listed in Additional file 5, Supplemental Table 5.

## Supplementary Information

**Additional file 1:** **Supplemental Figure 1.** Loss of mitochondrial green fluorescent protein (GFP) targeting in CMS-S pollen. a-l Spinning disc confocal micrographs of a-c normal (N) cytoplasm, young, bicellular pollen (YP); d-f CMS-S YP; g-i N-cytoplasm starch filling pollen (SFP); and j-l CMS-S collapsed pollen (CP). For each developmental stage, images of different pollen grains collected from the same anther are shown. a, d, g, j Bright field digital interference contrast (DIC). b, e, h, k Mitochondria-targeted GFP. c, f, i, l Hoechst nuclear staining. Bars = 20 µm for a, d, g, j; 5 µm for b, c, e, f, h, i, j, l

**Additional file 2:** **Supplemental Figure 2.** Full blot images of chemiluminescent immunoblots used for manuscript Fig. 7 (Immunodetection of mitochondrial proteins in developing maize microspores and pollen). Proteins (ATP1, ATP2, ATP 6, ATP8, mitochondrial ATP synthase subunits 1, 2, 6 and 8, respectively; COXII, cytochrome oxidase subunit 2; NAD7, NADH dehydrogenase subunit 7; AOX, alternative oxidase) and the PORIN loading control were immunodetected following denaturing gel electrophoresis and transfer to nitrocellulose membranes. Blot images were captured on a ChemiDoc ARS+ System with Image Lab^TM^ 6.1 software (Bio-Rad Laboratories, Hercules, CA). Three biological replicates (panels a-i, j-r, and s-a’) were performed for quantification of proteins by Image Lab^TM^. Boxed areas in panels a-i designate cropped regions of the blots that are shown in Fig. 7a. For each sample set, replicate blots were first decorated with antibodies against the PORIN loading control, processed and imaged. Blots were then decorated and processed with additional antibodies in succession. A third replicate blot was decorated with antibodies against AOX, which was not effectively separated from PORIN. Blots were not stripped between antibodies, and residual antibody signals are labeled in each panel. Red color indicates saturated pixels. No saturated exposures were used in quantification analysis. Protein samples 1-8 correspond to 1 ug of CMS-S immature ear mitochondrial protein, 1 ug of N-cytoplasm immature ear mitochondrial protein, 10 ug of total detergent soluble protein extracted from CMS-S microspore (MSP), N-cytoplasm MSP, CMS-S collapsed pollen, N-cytoplasm young pollen, N-cytoplasm starch filling pollen, and N-cytoplasm mature pollen, respectively. Protein molecular weight standards loaded in the far left lane of each blot were sometimes faintly labeled by the immunodetection reagents.

**Additional file 3:** **Supplemental Figure 3.** Mitochondrial transcript accumulation and editing in developing maize ears and pollen. a Denaturing gel electrophoresis and blot hybridization of mitochondrial RNAs from normal (N) or CMS-S (S) immature ear, microspore (MSP), or pollen stages: CP, collapsed pollen; SFP, starch-filling pollen; MP, mature pollen. Replicate blots of the ethidum bromide stained gel shown in the top panel were hybridized to full-length coding sequence probes labeled with the BrightStar® BioDetect™ system (Thermo Fisher). Transcripts were detected by exposure to X-ray film. Ethidium-stained mitochondrial ribosomal RNAs (*rrn26* and *rrn18*) and BrightStar-detected ATP synthase subunits 6 and 8 (*atp6* and *atp8*) are shown. b cDNA sequence traces showing all codon-changing RNA edits of *atp8* transcripts with minor amounts of partial editing at codon 20 in microspore (MSP) cDNAs. c Representative cDNA sequence traces of codon changing *atp6* transcript edits. No evidence of partial editing was observed for any codon changing edits in this transcript regardless of RNA source. MSP, microspore; CP, collapsed pollen; SFP, starch-filling pollen, MP, mature pollen.

**Additional file 4:** **Supplemental Table 1.** *atp6* transcript edits^a^. **Supplemental Table 2.** *atp8* transcript edits^a^.

**Additional file 5:** **Supplemental Table 3.** Antibodies for protein immunodetection. **Supplemental Table 4.** PCR reaction primers. **Supplemental Table 5.** Genbank accessions for cDNA sequences.

## Data Availability

All data are contained within the manuscript, supplemental materials and GenBank accessions: GU075810.1, GU075813.1, GU075811.1, GU075812.1, GU058049.1, GU058048.1, GU058047.1, GU05846.1 Antibodies and plant materials are available from the corresponding author upon request.

## Abbreviations

AOX: Alternative oxidase
ATP1, ATP2, ATP6, ATP8: ATP synthase subunits, 1, 2, 6, and 8, respectively
BCP: Bi-cellular pollen
CMS: Cytoplasmic male sterility
CMS-S: Cytoplasmic male sterility type S
COXII: Cytochrome oxidase subunit 2
CP: Collapsed pollen
DAPI: 4', 6-Diamidino-2-phenylindole
GFP: Green fluorescent protein
MP: Mature pollen (tricellular)
MSP: Microspore (uninucleate)
N: Normal cytoplasm
NAD7: NADH dehydrogenase complex subunit 7
PCD: Programmed cell death
ROS: Reactive oxygen species
SFP: Starch-filling pollen (bi-cellular and accumulating starch)
TUNEL: Terminal deoxynucleotidyl transferase dUTP nick end labeling
YP: Young pollen (bi-celluar with little starch)
