# Supplementary material for: Developmentally regulated mitochondrial biogenesis and cell death competence in maize pollen
Source: BMC Plant Biol. 2022 Nov 1;22:508. doi: 10.1186/s12870-022-03897-y (PMC9624016; doi:10.1186/s12870-022-03897-y)
Supplement: Supplementary file 1 — Additional file 1: Supplemental Figure 1. Loss of mitochondrial green fluorescent protein (GFP) targeting in CMS-S pollen. a-l Spinning disc confocal micrographs of a-c normal (N) cytoplasm, young, bicellular pollen (YP); d-f CMS-S YP; g-i N-cytoplasm starch filling pollen (SFP); and j-l CMS-S collapsed pollen (CP). For each developmental stage, images of different pollen grains collected from the same anther are shown. a, d, g, j Bright field digital interference contrast (DIC). b, e, h, k Mitochondria-targeted GFP. c, f, i, l Hoechst nuclear staining. Bars = 20 µm for a, d, g, j; 5 µm for b, c, e, f, h, i, j, l [file 12870_2022_3897_MOESM1_ESM.pdf]

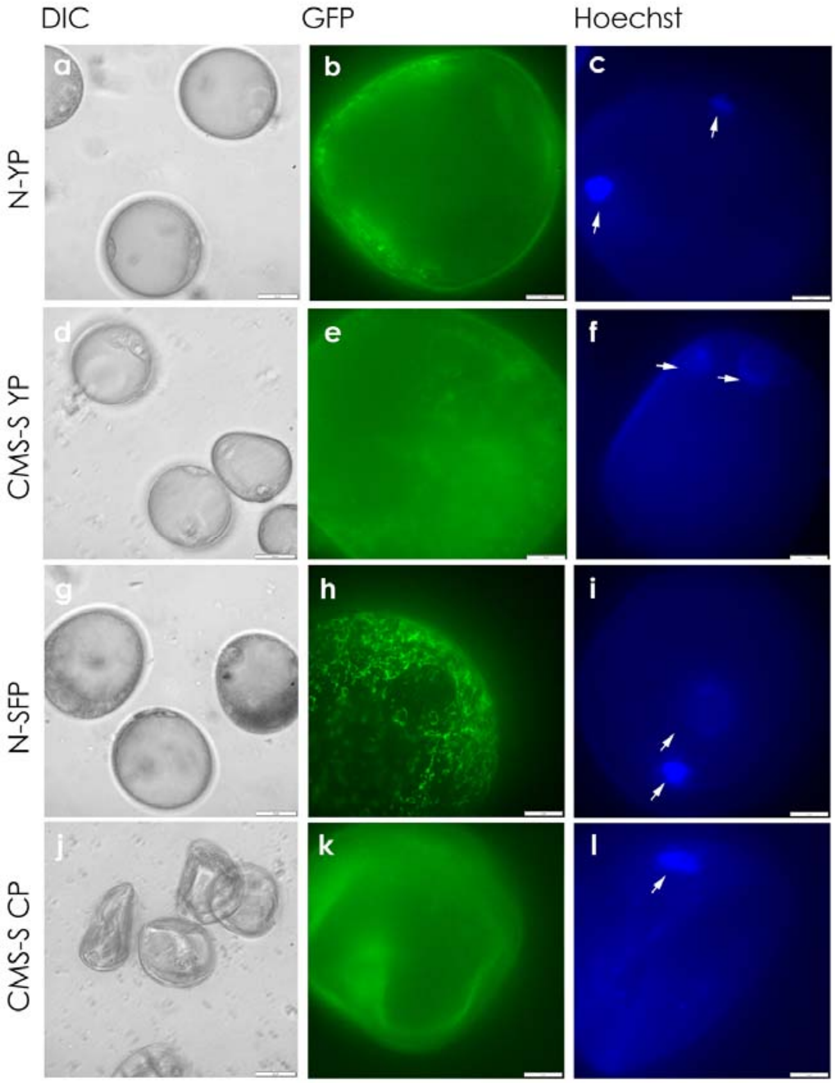

Supplemental Figure 1 Loss of mitochondrial green fluorescent protein (GFP) targeting in CMS-S pollen. **a-l** Spinning disc confocal micrographs of **a-c** normal (N) cytoplasm, young, bi-cellular pollen (YP); **d-f** CMS-S YP; **g-i** N-cytoplasm starch filling pollen (SFP); and **j-l** CMS-S collapsed pollen (CP). For each developmental stage, images of different pollen grains collected from the same anther are shown. **a, d, g, j** Bright field digital interference contrast (DIC). **b, e, h, k** Mitochondria-targeted GFP. **c, f, i, l** Hoechst nuclear staining. Bars = 20 µm for **a, d, g, j**; 5 µm for **b, c, e, f, h, i, j, l**
