# Supplementary material for: Developmentally regulated mitochondrial biogenesis and cell death competence in maize pollen
Source: BMC Plant Biol. 2022 Nov 1;22:508. doi: 10.1186/s12870-022-03897-y (PMC9624016; doi:10.1186/s12870-022-03897-y)
Supplement: Supplementary file 2 — Additional file 2: Supplemental Figure 2. Full blot images of chemiluminescent immunoblots used for manuscript Fig. 7 (Immunodetection of mitochondrial proteins in developing maize microspores and pollen). Proteins (ATP1, ATP2, ATP 6, ATP8, mitochondrial ATP synthase subunits 1, 2, 6 and 8, respectively; COXII, cytochrome oxidase subunit 2; NAD7, NADH dehydrogenase subunit 7; AOX, alternative oxidase) and the PORIN loading control were immunodetected following denaturing gel electrophoresis and transfer to nitrocellulose membranes. Blot images were captured on a ChemiDoc ARS+ System with Image LabTM 6.1 software (Bio-Rad Laboratories, Hercules, CA). Three biological replicates (panels a-i, j-r, and s-a’) were performed for quantification of proteins by Image LabTM. Boxed areas in panels a-i designate cropped regions of the blots that are shown in Fig. 7a. For each sample set, replicate blots were first decorated with antibodies against the PORIN loading control, processed and imaged. Blots were then decorated and processed with additional antibodies in succession. A third replicate blot was decorated with antibodies against AOX, which was not effectively separated from PORIN. Blots were not stripped between antibodies, and residual antibody signals are labeled in each panel. Red color indicates saturated pixels. No saturated exposures were used in quantification analysis. Protein samples 1-8 correspond to 1 ug of CMS-S immature ear mitochondrial protein, 1 ug of N-cytoplasm immature ear mitochondrial protein, 10 ug of total detergent soluble protein extracted from CMS-S microspore (MSP), N-cytoplasm MSP, CMS-S collapsed pollen, N-cytoplasm young pollen, N-cytoplasm starch filling pollen, and N-cytoplasm mature pollen, respectively. Protein molecular weight standards loaded in the far left lane of each blot were sometimes faintly labeled by the immunodetection reagents. [file 12870_2022_3897_MOESM2_ESM.pdf]

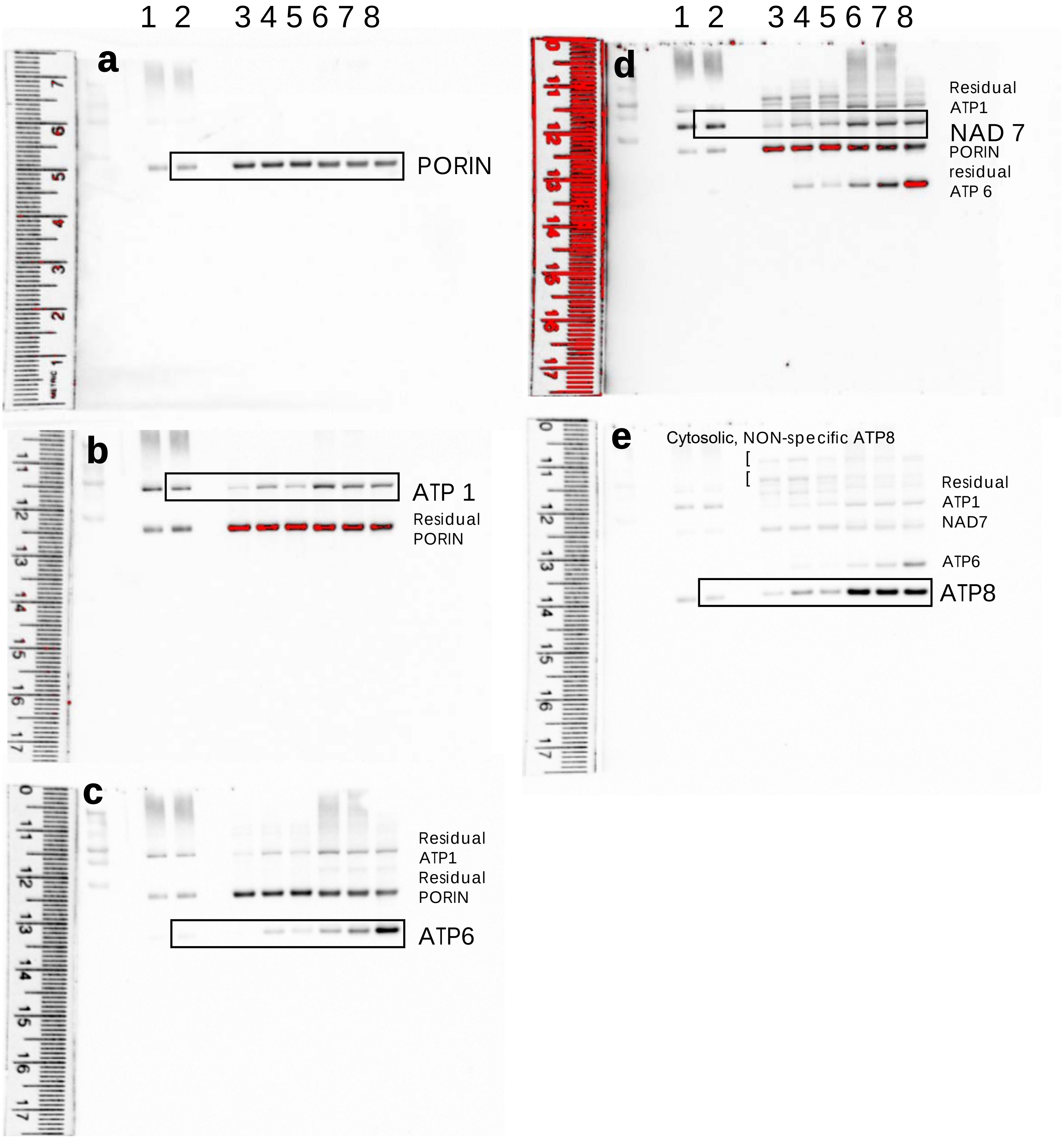
1 2 3 4 5 6 7 8
a
PORIN
b
ATP 1
Residual
PORIN
c
Residual
ATP1
Residual
PORIN
ATP6
1 2 3 4 5 6 7 8
d
Residual
ATP1
NAD 7
PORIN
residual
ATP 6
e
Cytosolic, NON-specific ATP8
Residual
ATP1
NAD7
ATP6
ATP8

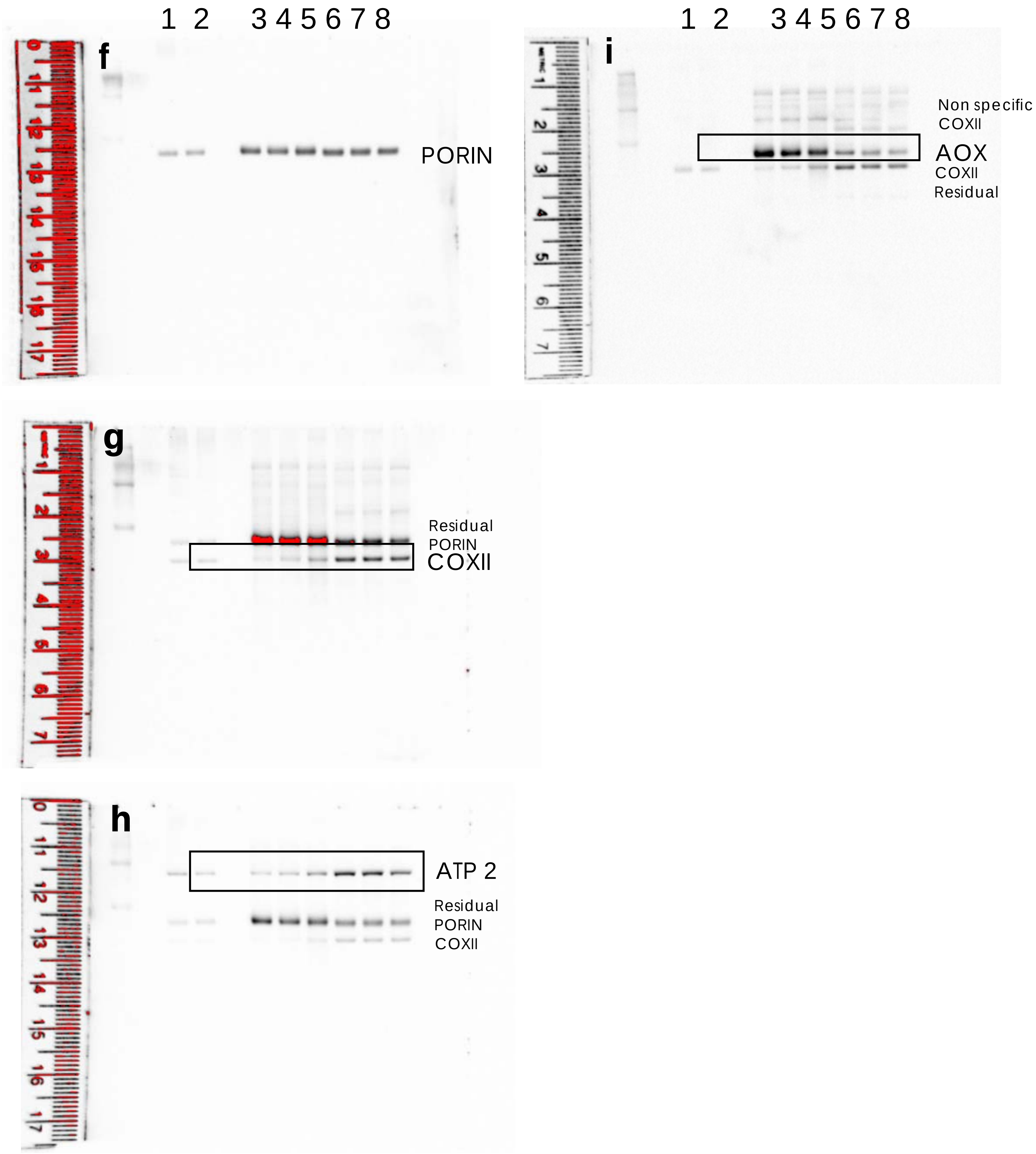
1 2 3 4 5 6 7 8
f
PORIN
i
Non specific
COXII
AOX
COXII
Residual
g
Residual
PORIN
COXII
h
ATP 2
Residual
PORIN
COXII

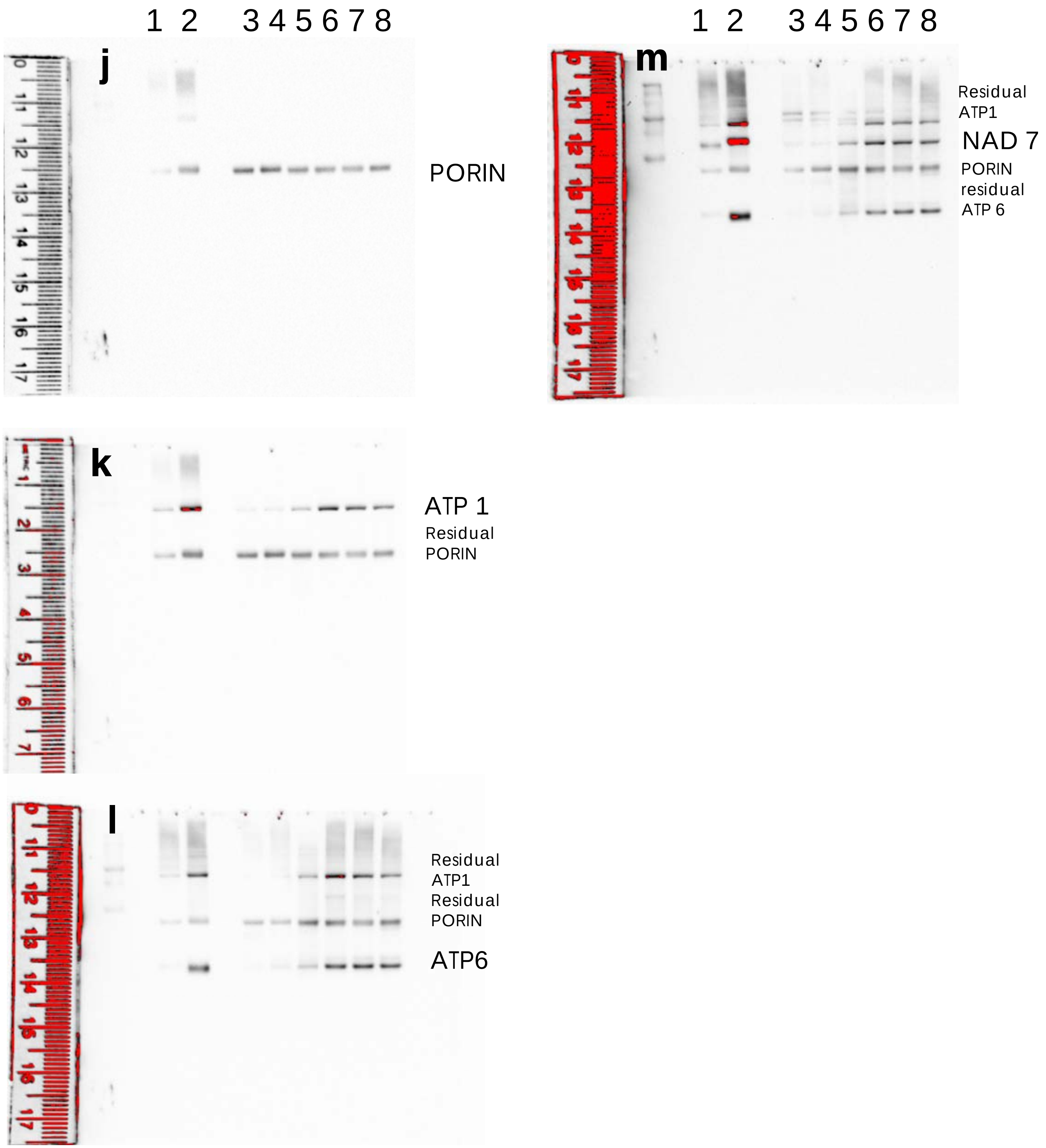
1 2 3 4 5 6 7 8
j
PORIN
1 2 3 4 5 6 7 8
m
Residual
ATP1
NAD 7
PORIN
residual
ATP 6
k
ATP 1
Residual
PORIN
l
Residual
ATP1
Residual
PORIN
ATP6

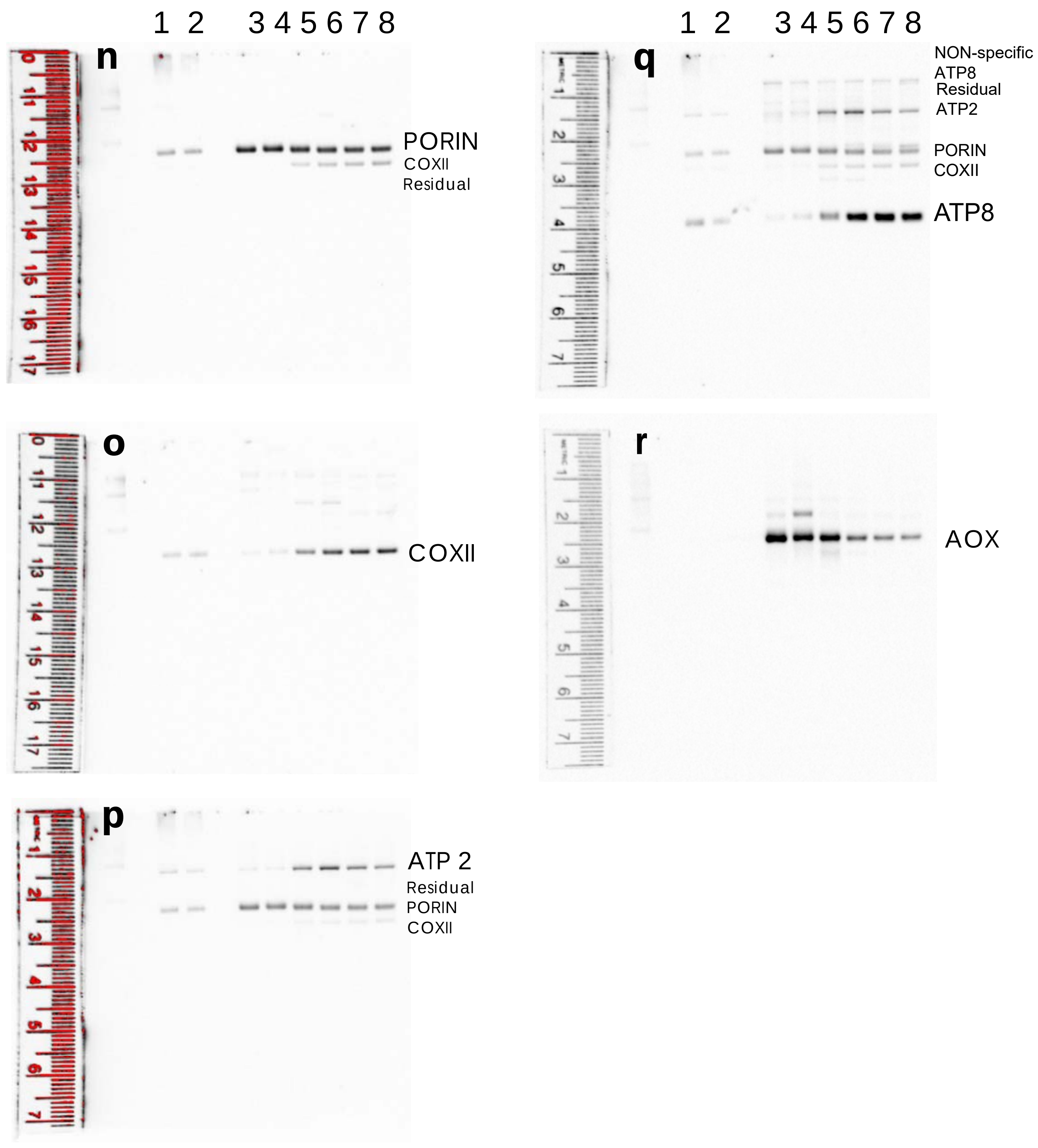
1 2 3 4 5 6 7 8
n
PORIN
COXII
Residual
o
COXII
p
ATP 2
Residual
PORIN
COXII
1 2 3 4 5 6 7 8
q
NON-specific
ATP8
Residual
ATP2
PORIN
COXII
ATP8
r
AOX

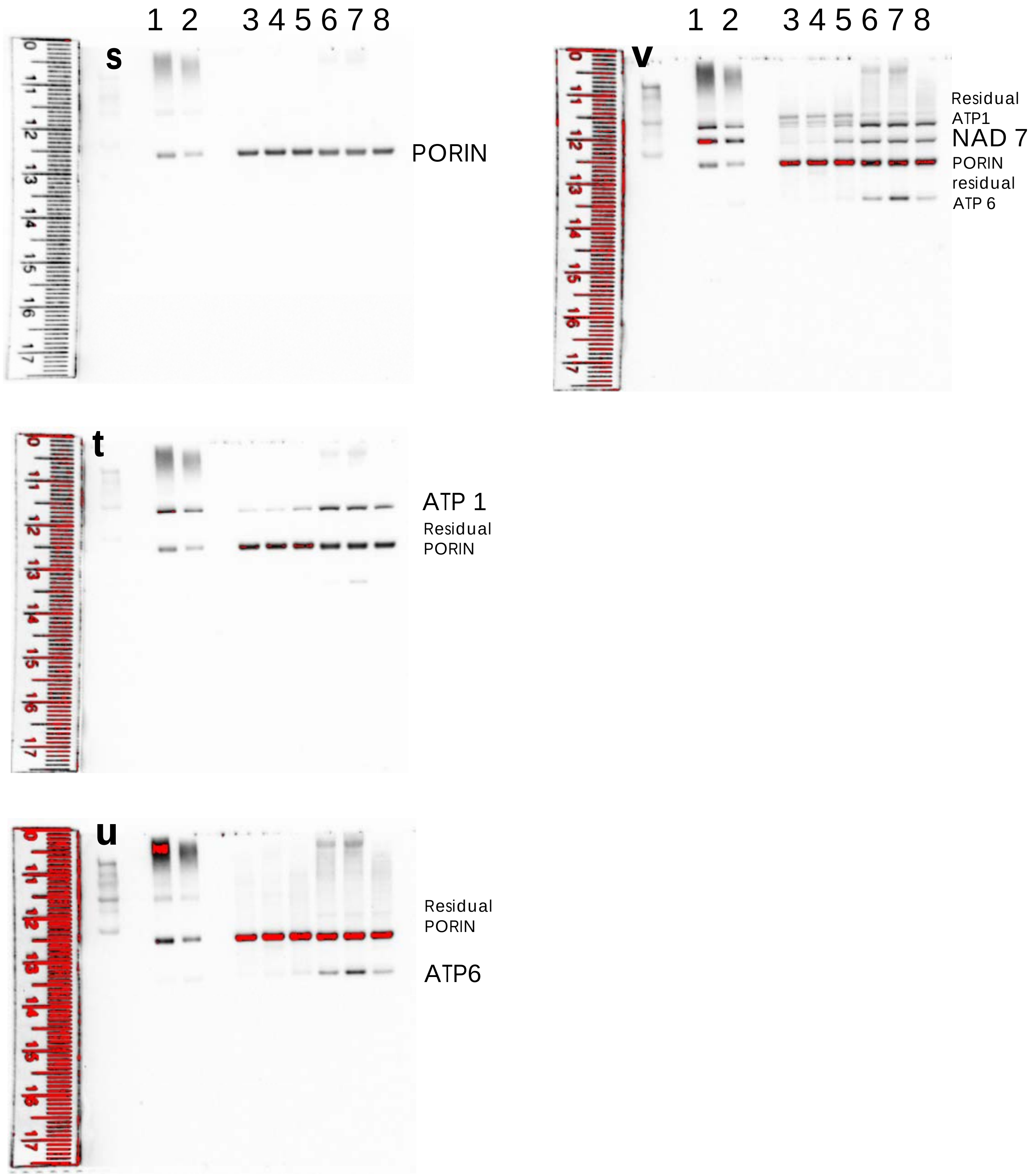
1 2 3 4 5 6 7 8
s
PORIN
t
ATP 1
Residual
PORIN
u
Residual
PORIN
ATP6
1 2 3 4 5 6 7 8
v
Residual
ATP1
NAD 7
PORIN
residual
ATP 6

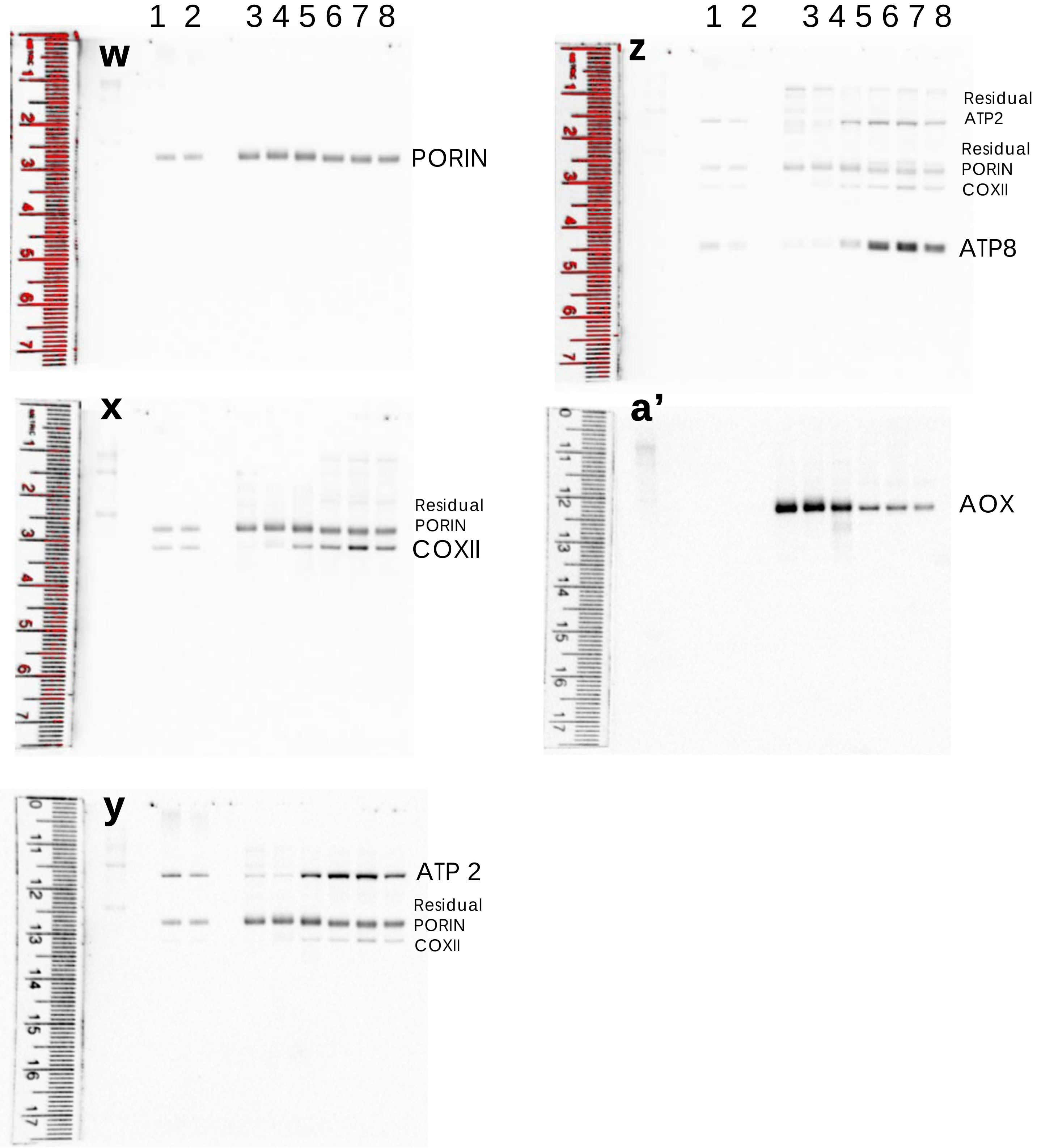
1 2 3 4 5 6 7 8
w
PORIN
1 2 3 4 5 6 7 8
z
Residual
ATP2
Residual
PORIN
COXII
ATP8
x
Residual
PORIN
COXII
a’
AOX
y
ATP 2
Residual
PORIN
COXII

Supplemental Figure 2 Full blot images of chemiluminescent immunoblots used for manuscript Fig. 7 (Immunodetection of mitochondrial proteins in developing maize microspores and pollen). Proteins (ATP1, ATP2, ATP 6, ATP8, mitochondrial ATP synthase subunits 1, 2, 6 and 8, respectively; COXII, cytochrome oxidase subunit 2; NAD7, NADH dehydrogenase subunit 7; AOX, alternative oxidase) and the PORIN loading control were immunodetected following denaturing gel electrophoresis and transfer to nitrocellulose membranes. Blot images were captured on a ChemiDoc ARS+ System with Image Lab™ 6.1 software (Bio-Rad Laboratories, Hercules, CA). Three biological replicates (panels a-i, j-r, and s-a') were performed for quantification of proteins by Image Lab™ . Boxed areas in panels a-i designate cropped regions of the blots that are shown in Fig. 7a. For each sample set, replicate blots were first decorated with antibodies against the PORIN loading control, processed and imaged. Blots were then decorated and processed with additional antibodies in succession. A third replicate blot was decorated with antibodies against AOX, which was not effectively separated from PORIN. Blots were not stripped between antibodies, and residual antibody signals are labeled in each panel. Red color indicates saturated pixels. No saturated exposures were used in quantification analysis. Protein samples 1-8 correspond to 1 ug of CMS-S immature ear mitochondrial protein, 1 ug of N-cytoplasm immature ear mitochondrial protein, 10 ug of total detergent soluble protein extracted from CMS-S microspore (MSP), N-cytoplasm MSP, CMS-S collapsed pollen, N-cytoplasm young pollen, N-cytoplasm starch filling pollen, and N-cytoplasm mature pollen, respectively. Protein molecular weight standards loaded in the far left lane of each blot were sometimes faintly labeled by the immunodetection reagents.
