# Supplementary material for: Developmentally regulated mitochondrial biogenesis and cell death competence in maize pollen
Source: BMC Plant Biol. 2022 Nov 1;22:508. doi: 10.1186/s12870-022-03897-y (PMC9624016; doi:10.1186/s12870-022-03897-y)
Supplement: Supplementary file 3 — Additional file 3: Supplemental Figure 3. Mitochondrial transcript accumulation and editing in developing maize ears and pollen. a Denaturing gel electrophoresis and blot hybridization of mitochondrial RNAs from normal (N) or CMS-S (S) immature ear, microspore (MSP), or pollen stages: CP, collapsed pollen; SFP, starch-filling pollen; MP, mature pollen. Replicate blots of the ethidum bromide stained gel shown in the top panel were hybridized to full-length coding sequence probes labeled with the BrightStar® BioDetect™ system (Thermo Fisher). Transcripts were detected by exposure to X-ray film. Ethidium-stained mitochondrial ribosomal RNAs (rrn26 and rrn18) and BrightStar-detected ATP synthase subunits 6 and 8 (atp6 and atp8) are shown. b cDNA sequence traces showing all codon-changing RNA edits of atp8 transcripts with minor amounts of partial editing at codon 20 in microspore (MSP) cDNAs. c Representative cDNA sequence traces of codon changing atp6 transcript edits. No evidence of partial editing was observed for any codon changing edits in this transcript regardless of RNA source. MSP, microspore; CP, collapsed pollen; SFP, starch-filling pollen, MP, mature pollen. [file 12870_2022_3897_MOESM3_ESM.pdf]

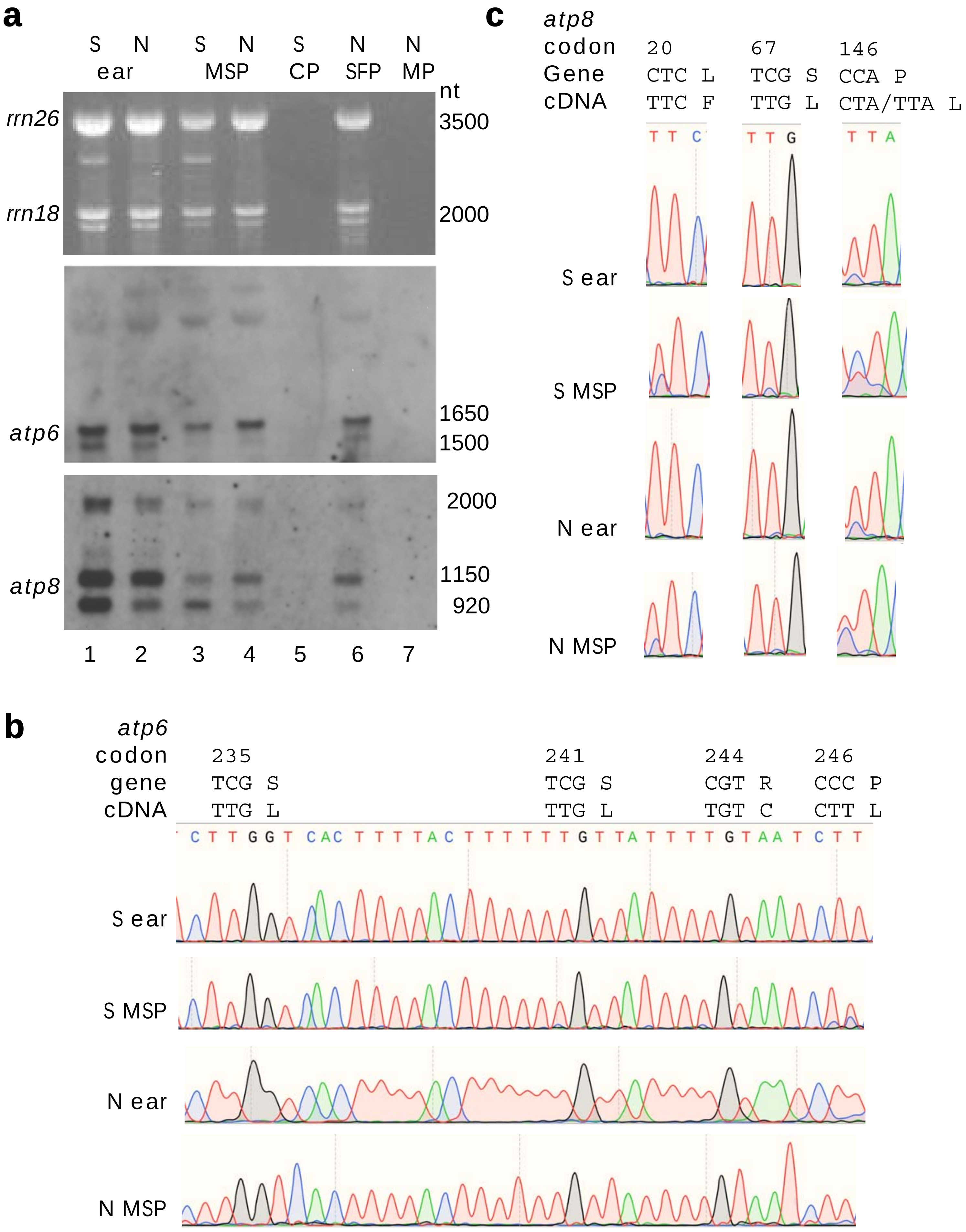
a
S N S N S N N
ear MSP CP SFP MP
nt
rrn26
3500
rrn18
2000
atp6
1650
1500
atp8
2000
1150
920
1 2 3 4 5 6 7
c
atp8
codon 20 67 146
Gene CTC L TCG S CCA P
cDNA TTC F TTG L CTA/TTA L
S ear
S MSP
N ear
N MSP
b
atp6
codon 235 241 244 246
gene TCG S TCG S CGT R CCC P
cDNA TTG L TTG L TGT C CTT L
S ear
S MSP
N ear
N MSP

Supplemental Figure 3. Mitochondrial transcript accumulation and editing in developing maize ears and pollen. a Denaturing gel electrophoresis and blot hybridization of mitochondrial RNAs from normal (N) or CMS-S (S) immature ear, microspore (MSP), or pollen stages: CP, collapsed pollen; SFP, starch-filling pollen; MP, mature pollen. Replicate blots of the ethidum bromide stained gel shown in the top panel were hybridized to full-length coding sequence probes labeled with the BrightStar® BioDetect™ system (Thermo Fisher). Transcripts were detected by exposure to X-ray film. Ethidium-stained mitochondrial ribosomal RNAs (*rrn26* and *rrn18*) and BrightStar-detected ATP synthase subunits 6 and 8 (*atp6* and *atp8*) are shown. **b** cDNA **s**equence traces showing all codon-changing RNA edits of *atp8* transcripts with minor amounts of partial editing at codon 20 in microspore (MSP) cDNAs. **c** Representative cDNA sequence traces of codon changing *atp6* transcript edits. No evidence of partial editing was observed for any codon changing edits in this transcript regardless of RNA source. MSP, microspore; CP, collapsed pollen; SFP, starch-filling pollen, MP, mature pollen.
