# Supplementary material for: Developmentally regulated mitochondrial biogenesis and cell death competence in maize pollen
Source: BMC Plant Biol. 2022 Nov 1;22:508. doi: 10.1186/s12870-022-03897-y (PMC9624016; doi:10.1186/s12870-022-03897-y)
Supplement: Supplementary file 4 — Additional file 4: Supplemental Table 1. atp6 transcript editsa. Supplemental Table 2. atp8 transcript editsa. [file 12870_2022_3897_MOESM4_ESM.docx]

Supplemental Table 1 *atp6* transcript edits^a^

| codon | gene sequence (amino acid) | cDNA sequence (amino acid) |
| --- | --- | --- |
| 172 | TCT (S) | TTT (F) |
| 195 | TCA (S) | TTA (L) |
| 212 | CCG (P) | CTG (L) |
| 235 | TCG (S) | TTG (I) |
| 241 | TCG (S) | TTG (I) |
| 244 | CGT (R) | TGT (C) |
| 246 | CCC (P) | CTC or CTT (L) |
| 311 | CAT (H) | TAT (Y) |
| 318 | TCA (S) | TTA (L) |
| 332 | TCA (S) | TTA (L) |
| 375 | TCA (S) | TTA (L) |
| 378 | CAT (H) | TAT (Y) |
| 380 | TCT (S) | TTT (F) |
| 392 | ACA (T) | ATA (I) |
| 393 | CAA (N) | TAA (stop) |

^a^ Editing sites determined by comparison of cDNA sequences amplified from CMS-S and normal (N) cytoplasm immature ears and microspores with CMS-S and N (NB type) cytoplasm mitochondrial genome sequences DQ490951.2 and NC_007982.1, respectively. The editing patterns of all four samples were identical.

Supplemental Table 2 *atp8* transcript edits^a^

| codon | gene sequence (amino acid) | cDNA sequence (amino acid) |
| --- | --- | --- |
| 20 | CTC (L) | TTC (F) |
| 67 | TCG (S) | TTG (L) |
| 146 | CCA (P) | TTA (L) |

^a^ Editing sites determined by comparison of cDNA sequences amplified from CMS-S and normal (N) cytoplasm immature ears and microspores with CMS-S and N (NB type) cytoplasm mitochondrial genome sequences DQ490951.2 and NC_007982.1, respectively. All codon-changing edits were made in all four samples, with minor traces of partial editing observed at codon 20 in both CMS-S and N cytoplasm microspores (Additional file 3 Supplemental Figure 3b).
