# Supplementary material for: Developmentally regulated mitochondrial biogenesis and cell death competence in maize pollen
Source: BMC Plant Biol. 2022 Nov 1;22:508. doi: 10.1186/s12870-022-03897-y (PMC9624016; doi:10.1186/s12870-022-03897-y)
Supplement: Supplementary file 5 — Additional file 5: Supplemental Table 3. Antibodies for protein immunodetection. Supplemental Table 4. PCR reaction primers. Supplemental Table 5. Genbank accessions for cDNA sequences. [file 12870_2022_3897_MOESM5_ESM.docx]

Supplemental Table 3 Antibodies for protein immunodetection

| Target  (kDa)^a^  Genome^b^ | Antigen | Antibody type^c^ | Source or Citation |
| --- | --- | --- | --- |
| AOX1/2  (34.0 & 36.0)^d^  N | Peptide: proprietary | RP | Agrisera AS04 054 |
| ATP1  (55.2)  M | Maize mitochondria | MM | Leuthy *et al.* 1993^e^ |
| ATP2  (52.2)  N | Maize mitochondria | MM | Leuthy *et al.* 1993^e^ |
| ATP6  (28.0)^f^  M | Peptide: SPLDQFGIHPILDLN | RP | Pring *et al.* 2006^g^ |
| ATP8  (17.7)  M | *E. coli* expression product:  NNNGILGISRILKLRNQLLSHRGNKIRSKDPNNLEDILRKGFSTGLSYMYSSLSEVSQWTVDYLGKRRKITLISDFGEISGSRGMERQILYLISKSSYNTSSSRITCWKNIMLTHVLHGQGSIIH6 | RP | Genscript custom antibody - present study |
| COXII  (29.7)  M | Peptide: proprietary | RP | Agrisera AS04 0530A |
| NAD7  (44.3)  M | Peptide: proprietary | RP | Abcam ab96160 |

^a^ The Protein Molecular Weight Calculator (http://www.bioinformatics.org/sms/prot_mw.html, accessed 04/25/2022) was used to predict the molecular weights of maize proteins based upon their predicted amino acid sequences minus, in the case of nuclear-encoded proteins, the predicted mitochondrial targeting sequences

^b^ M, mitochondria encoded; N, nucleus encoded

^c^ MM, mouse monoclonal antibody; RP, rabbit polyclonal antibody

^d^ Observed molecular weights of maize AOX2 and AOX3

Karpova OV, Kuzmin EV, Elthon TE, Newton KJ. Differential expression of alternative oxidase genes in maize mitochondrial mutants. Plant Cell. 2002;14:3271-84.

^e^ Lu B, Hanson MR. A single homogeneous form of ATP6 protein accumulates in petunia mitochondria despite the presence of differentially edited *atp6* transcripts. Plant Cell. 1994;12:1955-68.

^f^ Luethy MH, Horak A, Elthon TE. Monoclonal Antibodies to the [alpha]- and [beta]-Subunits of the Plant Mitochondrial F1-ATPase. Plant Physiol. 1993;101:931-7.

^g^  Pring DR, Tang HV, Chase CD, Siripant MN. Microspore gene expression associated with cytoplasmic male sterility and fertility restoration in sorghum Sex Plant Reprod. 2006;19:25-35.

Supplemental Table 4 PCR reaction primers

| Primer | Sequence | Purpose |
| --- | --- | --- |
| *atp6*F | 5’ccaagtctcttttgggagca | *atp6* forward flanking primer for cDNA amplification and sequencing |
| *atp6*iF | 5’TTCAATCCTTGGTGGAGCTT | *atp6* forward internal primer for cDNA sequencing |
| *atp6*R | 5’ggctcctcgtttttatgcaa | *atp6* reverse flanking primer for cDNA amplification and sequencing |
| *atp6*iR | 5’CCGGAAAGACCACCTATTTG | *atp6* reverse internal primer for cDNA sequencing |
| *atp8*F | 5’GAGGGTTGGTTTGATTGGAA | *atp6* forward flanking primer for cDNA amplification and sequencing |
| *atp8*R | 5’GGCAAGGATCCTCAGTCCTA | *atp6* reverse flanking primer for cDNA amplification and sequencing |

Supplemental Table 5 Genbank accessions for cDNA sequences

| Gene | Cytoplasm | RNA source tissues | Accession |
| --- | --- | --- | --- |
| *atp6* | N | immature ear | GU075810.1 |
| *atp6* | CMS-S | immature ear | GU075813.1 |
| *atp6* | N | microspore | GU075811.1 |
| *atp6* | CMS-S | microspore | GU075812.1 |
| *atp8* | N | immature ear | GU058049.1 |
| *atp8* | CMS-S | immature ear | GU058048.1 |
| *atp8* | N | microspore | GU058047.1 |
| *atp8* | CMS-S | microspore | GU05846.1 |
